# Nursing innovation: a comprehensive definition and framework for implementation

**DOI:** 10.1186/s12912-026-05124-7

**Published:** 2026-08-06

**Authors:** Kimberly A. Lewis, Jessica M. Phillips

**Affiliations:** Center for Nursing Excellence | Research, Evidence Based Practice, and Innovation Program, University of California, Los Angeles Health, 924 Westwood Blvd, Suite 720, Los Angeles, CA 90024 USA

**Keywords:** Innovation, Nursing innovation, Practice transformation, Concept analysis, Nursing leadership, Nursing administration, Nursing science, Nursing value, Human-centered design, Health innovation, Innovation typologies

## Abstract

**Background:**

Innovation has moved from the margins to the core of nursing’s professional identity. In the process, the profession has matured in its innovation competency level while the scope of influence has expanded across diverse domains. As a result, existing definitions and frameworks no longer capture the full breadth of nursing’s innovative functions and contributions. The purpose of this concept analysis was to systematically refine the definition and conceptual framework of nursing innovation to improve clarity.

**Methods:**

We conducted an expanded concept analysis of nursing innovation, combining a scoping review of definitions and typologies with Walker and Avant’s eight-step framework. Innovation literature was identified using a search of five electronic databases, historical documents, professional organization and government reports, and hand searches from 1850 to 2025. Data were extracted and synthesized from 50 sources (i.e., 39 peer-reviewed articles and books, 11 professional and government sources) to compare nursing, health, and general innovation definitions. These sources were then used to identify attributes, antecedents, consequences, and model cases.

**Results:**

The analysis yielded five defining attributes of nursing innovation that refine and clarify the concept: nursing agency, contextual novelty, purposeful orientation, implementation, and value creation. Nursing innovation was defined as the exercise of nursing agency to design, implement, and evaluate contextually novel and purposefully oriented approaches that create value and address challenges across the profession’s broad and expanding domains of influence. Antecedents and consequences were extracted at six levels, and 124 unique empirical referents were noted across the defining attributes. Model cases were presented for both traditional and non-traditional roles, innovation typologies, and settings. Gaps were identified in existing frameworks, and a divergence between nursing and general innovation paradigms was noted.

**Conclusions:**

This revised definition provides conceptual foundations for advancing nursing innovation while acknowledging the profession’s evolving influence in healthcare transformation. This analysis expanded the concept's boundaries beyond organizational settings, integrating the dual moral and economic imperatives that drive nursing innovation, and situating innovation across the novice-to-expert continuum. By clarifying attributes, typologies, and methods specific to nursing, it provides a novel framework that positions nursing innovation as a distinct, testable construct, establishing new directions for research, practice, and policy.

**Supplementary Information:**

The online version contains supplementary material available at https://doi.org/10.1186/s12912-026-05124-7.

## Background

Innovation has moved from the margins of nursing to the core of its professional identity [1]. The American Nurses’ Association (ANA), in the most recent scope and standards of practice, has charged nurses to lead and actively participate in innovation to transform health care and improve patient outcomes [1, 2]. Nursing innovation has been linked to increased job satisfaction, less workplace strain, organizational cost savings, workplace efficiency, value creation, and better patient outcomes [3, 4]. Together, these outcomes make nursing innovation not just desirable but essential to the future of health care.

As a profession, nursing has advanced its innovation competency beyond novice and advanced beginner to the competent stage. On Benner’s (1984) ladder, the competent stage reflects deliberate practice, structured approaches, and some sustained success, but not yet the consistent intuition or universal adoption of the expert stage [5]. Evidence of the competent stage can be seen in the establishment of dedicated nursing innovation centers and labs across academic and health settings, the inclusion of innovation in Magnet® designation standards [6, 7], the development of infrastructure and defined professional competencies within the ANA [1, 2], and the proliferation of accelerator programs and pitch events [1, 8–11]. However, innovation enculturation and individual competencies remain uneven across health systems and organizations, a finding consistent with the variability expected at the competent level of the novice-expert continuum [12].

Despite the growing imperative and professional maturation, the promise of nursing innovation has not yet been fully realized. Barriers such as systemic constraints, uneven preparation, and competing occupational demands impede the ability to consistently scale and sustain innovations. In parallel, as the profession expands its scope of influence and complexity beyond traditional domains, it is increasingly shaping fields such as venture capital and economics, private industry and entrepreneurship, legal affairs, public policy and elected offices, global advocacy, space travel, social media, communications, and marketing [9, 13–23]. This growth corresponds with a conceptual challenge: the frameworks and definitions historically used to guide nursing innovation were designed primarily for novices in traditional, supportive institutional contexts. Innovation is often positioned as dependent upon organizational endorsement and equated with nurtured creativity within medical centers or academia. As more nurses practice in non-traditional spaces and with higher levels of innovation expertise, these frameworks will no longer account for how innovation occurs beyond traditional institutional settings or without organizational scaffolding.

These challenges are compounded by an unsettled definition of nursing innovation that has been enthusiastically introduced, revised, and debated, but never fully stabilized [4, 24, 25]. Though widely invoked, the term was used inconsistently with definitions that sometimes conflate innovation with related terms like novelty, creativity, change, or invention. Further, when innovations occur in nursing, they may not be formally recognized and disseminated as such [26]. This can lead to a chronic under-estimation of nursing’s innovative impact, as well as under-diffusion and low visibility of nursing innovations [26].

Given these limitations, there is a need to revisit and refine nursing innovation. This paper provides an expanded concept analysis that delineates emerging innovation typologies while reconsidering the boundaries that have historically defined nursing innovation. Unlike earlier concept analyses that drew primarily on nursing studies, this analysis integrates insights from general innovation theory and healthcare innovation literature to derive a cross-disciplinary evidence base. This concept analysis aims to generate a measurable definition and framework attuned to the entire novice-to-expert continuum and applicable to diverse, traditional, and non-traditional professional contexts and roles. In doing so, this analysis seeks to better equip nursing education, practice, research, and policy with the conceptual clarity and theoretical tools necessary to steward the next stage of the profession’s innovation trajectory.

## Methods

### Design and guiding framework

We conducted a combined scoping review and concept analysis design to systematically map existing uses of *nursing innovation* and then refined and clarified defining attributes [27]. Following Arksey and O’Malley’s (2005) framework for scoping reviews [28], we conducted a comprehensive search of nursing, health, and interdisciplinary databases to identify peer-reviewed and gray literature that explicitly or implicitly engaged with the concept of innovation and related frameworks in nursing, health innovation, and general innovation theory. In contrast to a traditional concept analysis, the identification of essential characteristics was informed by a broader synthesis of diverse literature sources per the integrated scoping review methodology [27]. Search strings and keywords are detailed in Supplementary Table 1. Eligible sources included empirical studies, theoretical papers, policy documents, and professional guidelines. This approach captured both formal theoretical constructs and practical variations and emerging features.

To deepen clarity beyond mapping, we integrated the systematic, eight-step concept analysis process outlined by Walker and Avant (2019) [29]. The analysis proceeded through: (1) concept selection; (2) determination of the purpose; (3) identification of all uses of the concept; (4) specification of defining attributes; (5) construction of model, borderline, related, and contrary cases; (6) identification of antecedents and consequences; (7) identification of empirical referents; and (8) refinement of a working definition.

### Concept selection and determination of the purpose

Although the Walker & Avant (2019) approach cautions against profession-specific terms, we selected *nursing innovation* as the focal concept [29]. We intentionally retained the nursing qualifier because the literature treats nursing innovation as a distinct construct tied to professional scope and identity and is treated as analytically separable from general health innovation. The purpose of this analysis was to clarify and stabilize the concept for both traditional and non-traditional settings and roles by specifying the attributes and boundaries required for it to function as a measurable, theoretically coherent construct. A secondary purpose was to produce a refined framework to support its consistent application across scholarship and practice.

### Identification of all uses of the concept

#### Information sources and search strategy

**Fig. 1:**
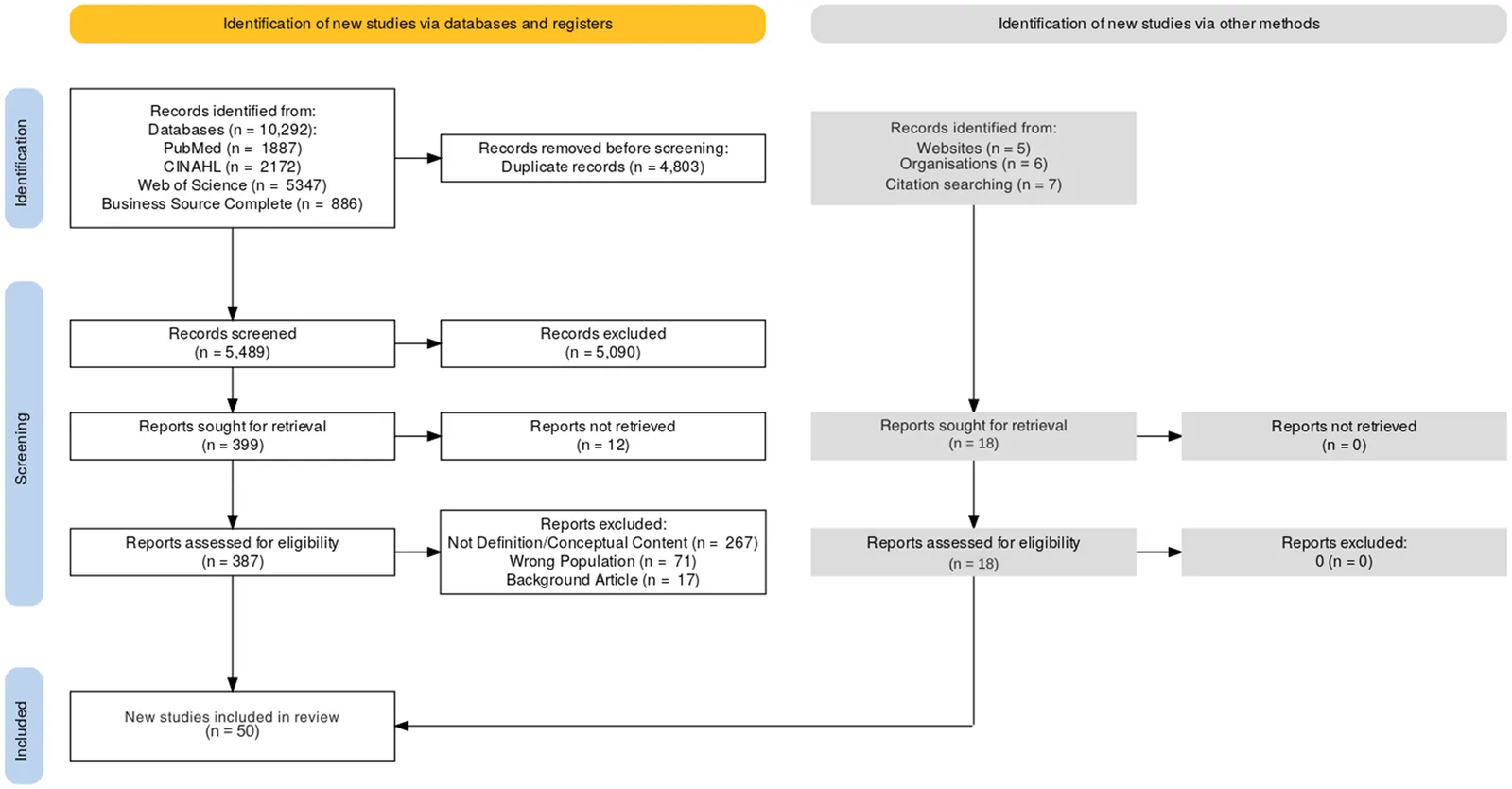
Literature search strategy – PRISMA flow diagram

The search strategy was detailed following the Preferred Reporting Items for Systematic Reviews and Meta-Analyses [30] guidelines in Fig. 1. Given that terms “innovation,” “health innovation,” and “nursing innovation” are often used interchangeably, have developed in parallel, and are influenced by many authorities beyond academia, we conducted a broad search that included dictionary definitions, governmental guidelines, professional guidelines, empirical studies, theoretical papers, policy documents, and historical works for any date through September 2025.

To compare definitions and define conceptual boundaries, we conducted a search across Cumulative Index of Nursing and Allied Health Literature (CINAHL), PubMed, Web of Science, online dictionaries, and Business Source Complete, supplemented by hand searches of digital sources. Using Boolean search terms and strings adapted per database, we used different combinations of vocabulary and key words for innovation with nursing specific terms and included articles that explicitly stated a definition or provided definitional clauses, scope notes, or operational frameworks. No language limits or date limits were applied at the search stage, though only articles available (i.e., written or translated) in English were retrieved. The results of each search were uploaded into Rayyan software to remove duplicates and organize the screening process [31].

Several innovation typologies did not surface reliably in the initial search; thus, we executed follow-on, domain-specific queries to capture sub-types of innovation relevant to nursing contexts. These included nursing-specific references to policy, marketing, economic or financial, social, communications, frugal, and education innovation.

#### Inclusion and exclusion criteria

Records were eligible if they contained a formal definition of innovation (general, health, or nursing), an innovation typology mapped to nursing contexts, or operational criteria that distinguished innovation from related constructs. We excluded items that used innovation rhetorically without definitional content. After removal of duplicates, a total of 50 sources (i.e., 39 peer reviewed journal articles and books, 6 professional sources, and 5 government sources) were eligible for inclusion in the final analysis.

#### Data extraction

For each included source, we extracted definition, concept type, and key characteristics/attributes, level (i.e., individual or organization/system level), antecedents and consequences, and empirical referents. Table 1 summarizes the data extracted from the 39 peer-reviewed journal articles and books and Table 2 summarizes the 11 professional and government sources. The extracted peer reviewed journal articles and books were grouped by nursing innovation, healthcare innovation, and general innovation theory. Nursing innovation articles included six foundational, five individual level, six organizational/system level, and five specialized context papers. The health innovation category included five foundational papers and one individual-level paper. The general innovation theory category included six foundational, four organization level, and two specialized context papers.

**Table 1:** Summary of included articles for nursing innovation, healthcare innovation, and general, cross-cutting innovation

Innovation Category	Innovation Subcategory	First Author et al. (Year)	Source Type	Definition and Concept Type	Key Characteristics
NURSING INNOVATION	Conceptual Foundations	Agryropoulos & Chronopoulou (2021) [32]	Conceptual Review	NURSING INNOVATION: introduction of a new concept, process, or product aimed at improving treatment, diagnosis, education, and research, with long0term objectives of improving quality, safety, and reducing costs. INNOVATION IN NURSING: the deliberate introduction and application of roles, groups, organization of ideas, new products and processes, which are designed to significantly benefit the individual, group, organization, or wider society.	Focus on educational and practice contexts Stresses adaptability, professional development, and patient-centered outcomes Innovation is both a process (teaching/learning strategies, care redesign) and product (new models, curricula, methods) Improve nursing practice, patient experiences, and care outcomes Innovation begins with a good idea but must be translated into something workable and feasible Benefits new knowledge and skills, scientific recognition of nurses, different work culture, higher incomes, improved patient safety, and quality of care Required supportive organizational culture (trust, openness, flexibility, tolerance of failure) Barriers include bureaucracy, burnout, negative leadership attitudes, resistance to change Nurse leaders create the culture, encourage lifelong learning, and facilitate innovation.
NURSING INNOVATION	Conceptual Foundations	Hughes (2006) [33]	Review Article	NURSING INNOVATION: Innovation inherently involves a new idea and may be defined differently for each organization into which it is introduced. More recent interpretations describe innovation as the conception, early adoption and implementation of significant new services, ideas or ways of doing things as government policy in order to improve or reform services, ideas and ways of doing things. INNOVATIVENESS: is the application of creative, new ideas and the implementation of inventions	Structured processes and mechanisms for encouraging creativity and opportunity to innovate; purposeful analysis of opportunities; achieve innovation within given environment; start small, simple, and focused; technologies/methods don’t have to be new to be innovative but must be new to the organization Traits of nurse innovators - high tenacity and determination, self-confidence, conscientiousness, ambition, desire for recognized qualifications, motivation to learn, perseverance, initiative, willingness to take risks and adopt new methods Systematic innovation requires overcoming barriers including short-term focus, lack of resources/staff, lack of systematic processes, high leadership turnover, and management incentives not rewarding innovation Successful innovations demonstrate knowledge brokering cycle: capturing good ideas, keeping ideas alive through communication, imagining new uses for old ideas, testing promising concepts; Examples span historical, research, clinical practice, business, education, technology, public health, and policy domains
NURSING INNOVATION	Conceptual Foundations	Kaya et al. (2015) [34]	Concept Analysis (Walker & Avant)	INNOVATION IN NURSING: Broadly, a new and different thing to do based on Latin root; in nursing, the process of developing and implementing new ideas, methods, approaches, technologies, or ways of working to improve care, meet needs, and adapt to changing conditions. Associated with novelty, creativity, and change. Applies to products, processes, practices, technologies, and organizational methods; can be radical or incremental, technical or administrative.	Innovation is both a product (new idea/method) and an ongoing process (implementation, adoption, improvement) - Attributes: novelty, utility, creativity, improvement, adaptability, and impact - Can be driven by need/gap, problem-solving, or proactive change - Incremental and radical types; technical and administrative forms - Requires vision, strategy, leadership, education, and an enabling organizational culture - Key for quality care, health promotion, disease prevention, and cost-effectiveness
NURSING INNOVATION	Conceptual Foundations	Lachman et al. (2009) [35]	Concept Analysis	NURSING INNOVATION: Application of creativity or problem solving that results in a widely adopted strategy, product, or service that meets a need in a new and different way. Innovations are about improvement in quality, cost-effectiveness, or efficiency.	Creativity, problem-solving, wide adoption, focus on improvement (quality, cost, efficiency), meeting recognized needs in a new/different way, implementation process emphasized.
NURSING INNOVATION	Conceptual Foundations	Rylee & Cavanagh (2023) [24]	Scoping Review	INNOVATION IN NURSING PRACTICE: Nursing innovation is identified as a significant departure from traditional nursing practices aimed at preventing, diagnosing, or treating disease in ways that improve care—beyond simply adopting new healthcare technologies or products. HEALTHCARE INNOVATION: defined as a significant departure from traditional practices to prevent, diagnose, or treat disease.	Three essential components for successful innovation: (1) ANTECEDENTS - Characteristics: Individual nurse (age, tenure, certification, education, motivation, risk-taking, flexibility, creative thinking, tenacity, self-confidence, analytical thinking, norm-breaking); Team (clear communication, cohesion, vision, mix of skills, trust, accountability, transparent leadership); Leader (entrepreneurial, risk-taking, flexible, supportive, quick thinking, foster inclusivity, autonomy, creativity, collaboration); Organization (creative climate, procedural justice, leadership, guidelines, learning opportunities, rewards, support staff, encourage inquiry, decision-making processes). (2) CULTURE - Individual/team participation, organizational vision alignment, avoiding mediocrity culture, job satisfaction, creative spaces, acceptance culture, leadership involvement, education about innovation value, adhocracy culture, risk-taking acceptance, trust, inquisitive spirit, ongoing education, resource provision. (3) PROCESS - Translation (adoption strategies, practice changes, education, communication, shared decision-making, management involvement); Assessment (feedback, adjustments, reinforcement, rigorous outcome measures); Outcome (sustaining vs disruptive innovation - sustaining builds on existing knowledge/experience, disruptive disturbs old systems/creates new markets); Sustainability (balance between stability and evolution, long-lasting benefits, monitoring outcomes, dynamic over time).
NURSING INNOVATION	Conceptual Foundations	Stout et al. (2023) [25]	Scoping Review	NURSE INVENTOR: may develop radical or incremental product innovations for patient, staff, or institutional benefit; notes lack of consensus definition and evolving professional structure; offers provisional definition: an altruistic practitioner who possesses the impetus, creativity, and knowledge to collaborate in the design and development of novel products or equipment for the betterment of patients, professionals, or healthcare institutions.	Four major themes: (1) Dynamic, multifactorial motivations including altruism, economic/institutional necessity, scientific advancement, and pursuit of clinical/technological excellence; (2) The nurse inventor role remains underdeveloped, lacking formal definition and integration; (3) Key facilitators include interdisciplinary collaboration (especially nurse-engineer partnerships), supportive institutions, positive leadership, and organizational culture; (4) Major barriers are lack of definition/integration into job structure, insufficient resources/funding, professional conformity, and perception of product invention as peripheral to nursing practice; strong call for further research and institutional culture change to support and operationalize this role.
NURSING INNOVATION	Individual Level (Nurses)	Asurakkody & Shin (2018) [36]	Concept Analysis (Walker & Avant)	INNOVATIVE BEHAVIOR: generating novel ideas and putting effort to implement them with confidence, overcoming possible challenges to produce new procedures, treatment strategies, or policies for restoring and health promotion of patient or clients	Eight defining attributes: (1) Opportunity exploration - identifying opportunities, discovery, search; (2) Idea generation - behavioral creativity, problem recognition; (3) Idea search - knowledge acquisition from existing sources; (4) Idea communication - effectively sharing ideas with managers; (5) Promotion of idea - persuading necessity and benefits; (6) Idea championing - confident implementation planning; (7) Application - developing, testing, commercializing; (8) Overcoming obstacles - adapting strategies for enhancement
NURSING INNOVATION	Individual Level (Nursing Leadership)	Gilmartin (1999) [37]	Review Paper	CREATIVITY AND INNOVATION IN NURSING LEADERSHIP: Creativity is positioned as the foundational element (“fuel”) for innovation in nursing organizations. The article defines creativity within organizations as being influenced by management practices in conjunction with creativity-relevant work group skills. Professional nurses are characterized as naturally creative in finding solutions for complex patient care needs, with the expansion of this creativity within organizational systems allowing for the evolution of professional nursing practice, improvement in care delivery, and enhanced organizational performance.	Primary management challenge is developing organizational cultures that value innovation, change, and creativity Adoption of an “ethic of innovativeness” Creative organizational behavior is a function of management practices in coordination with the skill level of individuals in the work group Professional nurses possess inherent creativity in finding solutions for complex patient care needs Expansion within systems enables evolution of professional nursing practice Focus on improvement in care delivery and organizational performance Emphasizes the critical role of organizational culture and leadership responsibility to create environments that support creative thinking and innovative solutions
NURSING INNOVATION	Individual Level (Nursing Students)	Heydari et al. (2023) [38]	Qualitative Study (Content Analysis)	INDIVIDUAL INNOVATION IN NURSING STUDENTS: The capacity for personal and professional dynamism, professional inventiveness, and integration of innovation drivers (mental/cognitive/psychological capabilities, internal/external stimuli, support networks). Defined as the active pursuit of new approaches, solutions, and adaptations in nursing by breaking routines, encouraging independence, embracing change/adaptation, and responding creatively to needs.	Three main themes: personal/professional dynamism, professional inventiveness (intellectual and practical), integration of innovation drivers Innovation driven by: mental, cognitive and psychological capabilities; internal stimuli (desire, interest, belief, talent); external stimuli (motivation, environment, teachability, support); support networks (family, university, policies, access to resources) Dynamic personality, independence, up-to-date knowledge, adaptation to change, teamwork/inter-professional skills, risk-taking, perseverance, problem-solving Necessity of supportive environments and organizational/managerial support Context-sensitive; influenced by sociocultural factors
NURSING INNOVATION	Individual Level (Nurses)	Leary et al. (2024) [39]	Quantitative Cross-sectional Survey & Factor Analysis	INNOVATIVENESS OF NURSES: Innovativeness is both a personal characteristic (a behavior dependent on perceived attributes of the innovation and an awareness of the need to innovate) and a measurable trait. Uses an adapted Scales for the Measurement of Innovativeness to quantify willingness to change and innovative behavior among US nurses engaging in innovation activities.	Innovativeness modeled as four factors: risk aversion, willingness to try new things (including influence/leadership), creativity/originality, and being challenged Nurses self-identifying as innovators had significantly higher innovativeness scores (121.3 vs. 112.9 out of 140) Key predictors: exposure to innovation/HCD/DT events, satisfaction with creative opportunities, institutional support, and participation in innovation events Both individual (risk attitude, creativity) and organizational factors (support, resources, protected time) are critical Majority are willing to innovate, but few feel they can always do so in daily work (barriers: time, resources, support)
NURSING INNOVATION	Individual Level (Nursing Leadership)	Snow et al. (2019) [40]	Review Article	CREATIVITY: Creativity is mental work to generate new ways of thinking; INNOVATION: innovation is the act of putting those creative results to use. INNOVATIVENESS: Innovativeness describes openness to new ideas, experimentation, risk-taking, and questioning current processes; it is a learnable competency, foundational for nurse leaders and essential for organizational transformation and competitive advantage in healthcare.	Emphasizes transformational leadership, workplace antecedents (organizational culture, trust, inquiry, learning, resource support), creative thinking tools, role modeling, risk-taking, empowerment through education, design thinking, multidisciplinary collaboration; promotes hands-on, sensory learning, and supportive environments for creative experimentation and innovation.
NURSING INNOVATION	Organization/System Level	Albert (2018) [8]	Implementation Framework	NURSING INNOVATION: the first practical, concrete implementation of an idea by or for Nursing that creates new knowledge; improves care, patient safety, and clinical outcomes; achieves efficiencies or administrative effectiveness; and allows for internal or external recognition to an individual, a department, or the clinical innovation center.	Foundational vs adjunct work; horizontal (elemental) and vertical (global) infrastructure required; involves failure tolerance; multiple pathways (serendipitous, incremental, methodical); requires multidisciplinary collaboration; needs structural support systems; cultural transformation from risk-averse to innovation-embracing
NURSING INNOVATION	Organizational/System Level	Barr et al. (2021) [10]	Qualitative Study (Thematic Analysis)	NURSING INNOVATION CENTERS: Organizational structures designed to create and sustain cultures of innovation through systematic approaches involving capacity building, engagement, leadership development, interprofessional collaboration, novel partnerships, and addressing diversity challenges to support nurse-led innovation and transformation	Four themes for creation: capacity building, faculty engagement, innovation structures/ecosystem, mentoring/support Six themes for sustainability: funding, engagement, leadership, interprofessional collaboration, novel partnerships, overcoming diversity innovation paradox; Innovation competency development; Process-oriented approach to building innovation capacity
NURSING INNOVATION	Organizational/System Level	Beaudet et al. (2023) [1]	Descriptive Article (ANA Programs)	INNOVATION FRAMEWORK (ANA): Based upon Lachman et al. (2006) definition of nursing innovation. Innovations are about improvement in quality, cost effectiveness, or efficiency and are cultivated via communities of practice.	Innovation competency framework: creativity, spanning boundaries, change management, considered risk-taking, failure tolerance; linked to nursing practice standards; emphasizes quality, cost-effectiveness, efficiency outcomes; supported by communities of practice and education Domains of their innovation communities of practice include: Business, Entrepreneurship, and Intrapreneurship; Data Science, AI, Augmented Intelligence; Education and Communication; New Care Delivery Models (Nursing and APRN Models); Planetary and Global Health; Policy and Determinants of Health Equity; Medical Technology and Devices
NURSING INNOVATION	Organizational/System Level	Gao et al. (2021) [41]	Quasi-Experimental Study (Innovation Competency Measurement)	INNOVATION COMPETENCY: defined as the ability to actively seek and develop new methods, new technologies and new tools to promote health, prevent diseases, improve the quality of care of patients, and apply innovation to work through teamwork and reasonable support channels with four elements: innovation subject, innovation process, innovation environment and pressure, and innovative products	Innovation competency measurement using two validated scales: (1) Scale of Clinical Nursing Staff Innovation Ability − 4 dimensions, 41 items (innovation subjects 17 items, innovation process 12 items, innovation environment/pressure 7 items, innovative products 5 items); (2) Scale of Nursing Scientific Research Ability − 6 dimensions, 30 items (research ideas, literature review, research design, conduct research, data analysis, report writing). Workshop intervention showed significant improvement, demonstrates measurable innovation competencies can be developed through systematic training
NURSING INNOVATION	Organizational/System Level	Joseph et al. (2019a) [11]	Qualitative Study & Theoretical Framework (Jobs to Be Done approach)	HEALTH CARE INNOVATION IN NURSING: New mindset enabling creative linkages to generate solutions or adaptations to practice/academic problems. Draws on the “Jobs to Be Done” theory: innovation is solution-focused progress to meet challenges, solve problems, or achieve goals in practice, education, research, or system processes. Emphasizes interconnectedness between academic and clinical settings and the need for theory-driven academic-practice partnerships.	Theoretical framework links context, challenges, opportunities, innovation types, and impacts across academic and practice settings Highlights interconnected challenges (patient care, education, health system) and multi-domain innovation types (care delivery, interventions, transitions, research/translation, collaboration, tech/data, teaching, process improvement) Leadership, organizational support, workplace relationships, and collaborative partnerships foster innovation capacity and scaling Workarounds, nurse-driven insight, and shared “maker spaces” as sources of innovation Advocates reward, incentive, and support systems plus cross-boundary partnerships for sustainable and impactful nursing innovation
NURSING INNOVATION	Organizational/System Level	Slettebø et al. (2021) [42]	Qualitative ethnographic study	SOCIAL INNOVATION: process of encompassing the emergence and adoption of socially creative strategies, which reconfigure social relations in order to actualize a given social goal. PUBLIC SECTOR INNOVATION: new ideas that work at creating public value	Innovation is social, collective, and oriented to improving lived experience rather than purely efficiency or technology; Innovation is relational and process based rather than product-based. 5 Subthemes: opening to the surroundings, expanding and strengthening community of practice, facilitating customized activities, ensuring sufficient nutrition and enjoyable mealtimes, preventing unrest and disturbing behavior Must be in part new rather than improvements, and must be taken up (not just good ideas), and have to be useful Grounded in person-centered care Emphasizes end-user participation, culture co-creation, staff collaboration, and structural support Culture change toward openness, inclusivity, and community engagement End user dignity, agency, and participation in social life
NURSING INNOVATION	Typology Detail	Joseph et al. (2019b) [43]	Qualitative study	INNOVATION: new mindset triggered by a different context to enable new, useful, creative, and unexpected linkages that will generate a solution or adaptation to a practice, scientific teaching, or research problem that adds value to the university or to healthcare. INNOVATIVENESS: social interactions in practice and education that generate, accept, and implement new processes, products, or services within organizations. INNOVATION WORK BEHAVIOR: initiation, championing, and implementation of new ideas to improve performance	Eight innovation typologies identified: 1. Care delivery models 2. Process transformation 3. Patient care interventions 4. Research and translational methods 5. Communication and collaboration 6. Technology and data use 7. Role transitions 8. Teaching methods
NURSING INNOVATION	Typology Detail: Frugal Innovation Method	Stephens et al. (2022) [44]	Review paper	FRUGAL INNOVATION: low cost and efficacious, new or adapted products or services, mostly emerging from contexts of institutional voids and resource constraints, involving the creative use of existing resources; a flexible approach to thinking about repurposing and sustainable resourcing.	Low-cost, efficacious solutions that creatively repurpose existing resources Originates in low- and middle-income countries due to resource constraints, but has growing relevance in high-income settings Includes approaches such as jugaad, reverse innovation, and Gandhian innovation (doing more with less for more) Categories of frugal innovation: Lean tools and techniques (simplification) Opportunistic solutions Contextualized adaptations Bottom-up innovations Barriers: limited visibility in high-income country research, lack of standardized reporting, challenges scaling into formal clinical guidelines
NURSING INNOVATION	Typology Detail: Policy Innovation Organizational/System Level	Younas (2025) [23]	Narrative Review	POLICY IMPLEMENTATION SCIENCE: Implementation science is the systematic process of identifying, applying, and evaluating context-specific approaches to translate evidence and innovation into practice—including both interventions and policies. Policy implementation science is specifically concerned with identifying and utilizing strategies to support the uptake and integration of policy into nursing and health practice, focusing on the practical translation of evidence-informed and organizational policies into action. Policy can be conceptualized as something to adopt, something to implement, a context to understand, or a strategy to adapt for optimal implementation.	Distinguishes four ways policy is conceptualized: as something to adopt, implement, context to understand, or as a strategy for facilitating other interventions. Policy innovation is dependent upon addressing the policy-practice gap Necessity for clarity in policy definition, engagement of policymakers throughout the process, and methodological pluralism (mixed/multimethod approaches) for real-world complexity. Calls for proactive nursing leadership and research engagement in policy implementation to create meaningful and sustainable system change and impact. Discusses the importance of timely, context-aware, and sustainable policy rollout in nursing, and proposes recommendations for advancing the field.
NURSING INNOVATION	Typology Detail: Process/Technology Innovation	Bahari et al. (2021) [45]	Grounded Theory Study (Caring Process as Technology Innovation Method)	TECHNOLOGICAL CREATIVITY: the creative process of co-creating nursing technologies with nurse-innovators and a network of collaborators in a supportive environment, to express caring for patients, to improve the quality of nurse’s caring, and for the ultimate goal of achieving patient wellness	Seven-phase process: investigating, analyzing, designing, prototyping, testing, applying, monitoring; Requires nurse-innovators with three characteristics: thinking outside the box, positive emotional engagement, team synergy; Involves collaboration and supportive environment; Driven by caring for patients, nurses, management, and achieving patient wellness
NURSING INNOVATION	Typology Detail: Service/Role Innovation	Roberts-Davis et al. (1998) [46]	Empirical Study with Typology Development	NURSING ROLE INNOVATON: nursing roles that extend or advance practice through additional preparation, competence, autonomy, and new domains of activity that move beyond conventional role boundaries.	Appropriate preparation, competence in practice, advanced level of practice, autonomy within nursing scope. Expansion of roles into new domains (skill/task, role, condition, area, client group, generic individual, generic community) Characterized by specialist plus functions that include leadership, breaking new ground, management, education, and research. Often extended into areas previously reserved for medicine or other professionals.
HEALTHCARE INNOVATION	Conceptual Foundations	Flessa & Huebner (2021) [47]	Conceptual Framework with Case Studies	HEALTHCARE INNOVATION FRAMEWORK: Innovation can be seen as the initial commercial implementation of a new idea as well as the economic optimization of knowledge utilization in healthcare. In the health care sector, innovations are the source of any improvement in the quality of services and quality of life, but also a steady challenge to existing health care providers and systems	Comprehensive 5-dimensional typology framework: (1) Object (product/service/process/social); (2) Relation to standard (competing/enriching); (3) System level (micro/meso/macro); (4) Extent of change (incremental/radical/disruptive); (5) Technology readiness level (TRL 1–9). Four-promoter adoption model: expert, power, administrative, relationship promoters. Meta-stability concept: artificially stabilized dysfunctional systems. Three case studies: personalized medicine, digital health, implants. Systematic innovation management approach
HEALTHCARE INNOVATION	Conceptual Foundations	Greenhalgh et al. (2004) [48]	Systematic Review & Conceptual Model	DIFFUSION OF INNOVATION IN HEALTH SERVICES: A novel set of behaviors, routines, or ways of working that are directed at improving health outcomes, efficiency, cost, or experience, and are implemented via planned and coordinated actions. Distinguishes diffusion (passive spread), dissemination (active persuasion/efforts), implementation (mainstreaming), and sustainability (routine until obsolete). Synthesizes 13 research traditions and proposes a unified multi-level conceptual model for diffusion and implementation in service organizations.	Multi-level model: innovation attributes, adoption/assimilation, communication/influence, organizational/system context, interorganizational/network, implementation, outer context 13 research traditions synthesized (sociology, communication, marketing, org studies, EBM, knowledge management, complexity, etc.) Key innovation attributes: relative advantage, compatibility, complexity, trialability, observability, reinvention, risk, ‘fuzzy boundaries’, task relevance, required knowledge, support/augmentation Adoption as an active, interpretive, socially contingent process; Assimilation non-linear, interactive, iterative; often not sequential Social networks, homophily, opinion leaders, champions, boundary spanners are influential Org innovativeness: structure (size, resources, decentralization), culture, absorptive capacity, leadership, risk climate, data, knowledge sharing System readiness: tension for change, fit, resources, support, feedback loops; Implementation & sustainability: leadership, communication, resources, HR/training, routinization, feedback, adaptation Advocates process-oriented, ecological, multi-method, theory-driven, participatory research in organizational innovation.
HEALTHCARE INNOVATION	Conceptual Foundations	Omachonu & Einspruch (2010) [49]	Conceptual Framework & Review	HEALTHCARE INNOVATION: the introduction of a new concept, idea, service, process, or product aimed at improving treatment, diagnosis, education, outreach, prevention and research, with long-term goals of improving quality, safety, outcomes, efficiency, and costs.” Also presents multi-dimensional framework: product, process, marketing, organizational, and structural innovation.	Product, process, organizational, marketing, and structural innovation types. Operational dimensions (outcomes, efficiency, quality, cost, safety, aging population, workforce shortages, patient satisfaction, profitability). Environmental dimensions (organizational culture, leadership, regulatory/physician acceptance, partnerships, complexity). Key stakeholders (patients, providers, organizations, innovators, regulators) with distinct needs. Innovation process is typically: problem identification, idea generation, evaluation, development, first use, commercialization, diffusion. Innovation can be incremental (nondisruptive/sustaining) or disruptive (radical/revolutionary/transformational). Information technology is a major driver. Challenges include diffusion/adoption, stakeholder alignment, regulatory constraints, culture, dissemination. Emphasizes multidimensionality and systems thinking for innovation analysis.
HEALTHCARE INNOVATION	Conceptual Foundations	Thakur et al. (2012) [50]	Qualitative Study (C-level Interviews & Literature Review)	HEALTHCARE INNOVATION: the adoption and implementation of best-demonstrated practices proven to be successful, ensuring patient safety, best outcomes, and improved organizational performance; innovation helps practitioners work smarter, faster, better, and cost-effectively. Draws on multiple healthcare executive perspectives and integrates strategic, process, and technology views.	Focus on practical change and continuous improvement; influenced by internal (organizational culture, efficiency, leadership) and external (market, regulation, competition) environments; major role of IT/IS in strategy and rollout; bottom –up information sharing and employee engagement; innovation seen as new service lines/responding to uncertainty; iterative model: idea generation, decision, rollout, evaluation, modification; emphasizes evaluation, adaptation, and collaboration across organizational levels; future trends highlight IT, outcome-based management, and reforms.
HEALTHCARE INNOVATION	Conceptual Foundations	Weberg (2009) [51]	Concept Analysis	HEALTHCARE INNOVATION: something new, or perceived as new by the adopting population, that can drive change and redefine healthcare’s economic or social potential.	Innovation is distinguished from invention, idea generation, diffusion, and routine novelty because it requires implementation, contextual uptake, and consequences within a population, organization, or system. Emphasizes innovation as a nonlinear process shaped by leadership, financial support, idea generation, teamwork, culture, and demand. Draws from business, economics, nursing, medicine, diffusion, and healthcare literature. Identifies newness, relative advantage, compatibility, observability, trialability, reduced perceived risk, and fit with culture as important features of innovation. Frames healthcare innovation as both social and economic, with consequences including new processes, policies, organizational structures, outcomes, and system change.
HEALTHCARE INNOVATION	Individual Level (Allied Professionals and Dietitians)	Hewko (2022) [52]	Quantitative research study	INNOVATION: an employee’s intentional introduction or application of new ideas, behaviors, products, processes, and procedures to his or her work role, work unit or organization ROLE INNOVATION: redefining or approaching one’s role differently than predecessors or peers	Innovation conceptualized as work behavior Individualized predictors: personality traits (conscientiousness, openness), personal growth initiative, voluntary professional association memberships Job specific predictors: motivation to innovate, perceived favorability of innovation in performance evaluations. Organizational predictors: leadership, culture, social capital, communication, resources, organizational slack Individual level factors, especially role innovation and conscientiousness, were the strongest predictors or innovative output; org and job-specific factors played smaller roles
GENERAL INNOVATION THEORY	Conceptual Foundations	Crossan & Apaydin (2010) [53]	Systematic Literature Review & Framework	ORGANIZATIONAL INNOVATION FRAMEWORK: production or adoption, assimilation, and exploitation of a value-added novelty in economic and social spheres; renewal and enlargement of products, services, and markets; development of new methods of production; and establishment of new management systems. It is both a process and an outcome	Multi-dimensional framework with three meta-constructs: (1) Innovation Leadership (individual/group level with upper echelon theory); (2) Managerial Levers (organizational level with dynamic capabilities - strategy, resources, structure, culture, learning); (3) Business Processes (process level - initiation, development, project management, commercialization, portfolio management); Innovation as both process (how) and outcome (what) with 10 dimensions Systematic review of 524 papers over 27 years
GENERAL INNOVATION THEORY	Conceptual Foundations	Garcia & Calantone (2002) [54]	Literature Review & Typology Framework	TECHNOLOGICAL INNOVATION TYPOLOGY: Innovation is an iterative process initiated by the perception of a new market and/or new service opportunity for a technology-based invention which leads to development, production, and marketing tasks striving for the commercial success of the invention (OECD 1991). Product innovativeness is a measure of the potential discontinuity a product can generate in the marketing and/or technological process with three types: RADICAL (macro/micro marketing and technology discontinuities), REALLY NEW (macro discontinuity in either marketing OR technology + micro discontinuities), INCREMENTAL (micro-level discontinuities only)	Comprehensive 3-type typology framework resolving 15 constructs and 51 scale items from 21 empirical studies. RADICAL INNOVATIONS (12.5%): Discontinuities in both macro/micro levels and marketing/technology S-curves, create new industries/markets. REALLY NEW INNOVATIONS (50%): Macro discontinuity in either marketing OR technology + micro discontinuities, new product lines/market extensions. INCREMENTAL INNOVATIONS (37.5%): Micro-level only discontinuities in marketing and/or technology, product improvements/modifications. Uses Boolean logic classification system. Technology/Marketing S-curve analysis for identification. Second-order factor model for operationalization
GENERAL INNOVATION THEORY	Conceptual Foundations	Rogers (2003) [55]	Scholarly Monograph (Diffusion of Innovations)	INNOVATION: An innovation is an idea, practice, or object that is perceived as new by an individual or other unit of adoption. The perceived newness of the idea for the individual determines his or her reaction to it. DIFFUSION: Diffusion is the process by which an innovation is communicated through certain channels over time among the members of a social system.	Four main diffusion elements: innovation, communication channels, time, social system Five innovation attributes influencing rate of adoption: relative advantage, compatibility, complexity, trialability, observability Diffusion as special type of communication, centering on uncertainty reduction through information exchange Innovation-decision process: knowledge, persuasion, decision, implementation, confirmation S-shaped adoption curve; adopter categories: innovators, early adopters, early majority, late majority, laggards Social system factors: norms, opinion leaders, change agents, types of innovation-decision (optional, collective, authority, contingent) Organizational implementation and consequences, including unanticipated effects and equity implications
GENERAL INNOVATION THEORY	Conceptual Foundations	Schumpeter (1934) [56]	Book (Schumpeterian Theory)	INNOVATION FRAMEWORK (Schumpeterian Theory): Innovation is the practical implementation of inventions - ‘as long as they are not carried into practice, inventions are economically irrelevant’ with NEW COMBINATIONS: Five cases - (1) new good/quality; (2) new production method; (3) new market; (4) new supply source; (5) new industry organization	Economic innovation framework: Innovation vs invention distinction (practical application vs discovery); New combinations framework (5 cases); Creative destruction process; Entrepreneurial innovation (entrepreneurs as innovators, not inventors); Innovation as endogenous factor disrupting economic equilibrium; Economic development driven by innovation
GENERAL INNOVATION THEORY	Conceptual Foundations	Taylor (2017) [57]	Literature Review & Theoretical Model	INNOVATION: Innovation is the creative process whereby new or improved ideas are successfully developed and applied to produce outcomes that are practical and of value, integrating creativity, implementation, novelty, and value. Composite definition synthesized from a review of academic, public sector, and government models and definitions.	Multidimensional: new products/services or improvements on existing ones; strong link to creativity (creative process and academic models); practical and value-driven outcomes. Examines classic and contemporary creative process models, highlighting preparation, incubation, illumination/ideation, evaluation, implementation. Applied to social/public service with typology covering total, evolutionary, expansionary, and developmental innovation
GENERAL INNOVATION THEORY	Organizational/System Level	Baregheh et al. (2009) [58]	Content Analysis Study	ORGANIZATIONAL INNOVATION: Innovation is the multi-stage process whereby organizations transform ideas into new/improved products, service or processes, in order to advance, compete and differentiate themselves successfully in their marketplace	Six key attributes: (1) Nature of innovation - form (new/improved) (2) Type of innovation - kind (product/service/process/technical) (3) Stages of innovation - steps from idea generation to commercialization (4) Social context - entities/systems/people involved (5) Means of innovation - necessary resources (technical/creative/financial) (6) Aim of innovation - overall organizational results desired
GENERAL INNOVATION THEORY	Organizational/System Level	Budden & Murray (2019) [59]	Working Paper (MIT Framework)	INNOVATION FRAMEWORK (MIT): MIT Innovation Initiative - process of taking ideas from inception to impact (with distinction between big “I” Innovation − 10x transformation and little “I” innovation − 10% improvement)	Innovation ecosystem framework: Innovation spectrum from little “i” (10% improvement) to big “i” (10x transformation) Problem/solution matrix Innovation ecosystem with twin engines: Innovation Capacity (I-Cap) and Entrepreneurial Capacity (E-Cap) Five stakeholders: Government, Corporates, Universities, Entrepreneurs, Risk Capital providers
GENERAL INNOVATION THEORY	Organizational/System Level	Drach-Zahavy & Somech (2001) [60]	Empirical Study (Quantitative Survey)	TEAM INNOVATION: the intentional introduction and application within a team, of ideas, processes, products or procedures new to the team, designed to significantly benefit the individual, the team, the organization, or wider society	Emergent (bottom-up) innovation mediated by four team interaction processes: (1) Exchanging information - sharing knowledge and experience (2) Learning - reflecting on objectives, strategies, processes (most potent predictor) (3) Motivating - cognitive commitment to goals (4) Negotiating - mutual influence and opinion expression Requires both team structures (functional heterogeneity, meeting frequency) and social processes Intentional and application-focused with social element
GENERAL INNOVATION THEORY	Organizational/System Level	Klein & Sorra (1996) [61]	Theoretical Model & Literature Review	INNOVATION IMPLEMENTATION: The process of gaining employees’ appropriate and committed use of an innovation. Implementation effectiveness (quality/consistency of use) is a function of organizational climate for implementation and the fit of innovation to users’ values. Defines implementation (distinct from adoption), implementation climate, innovation-values fit, and implementation effectiveness (use quality/consistency), with outcomes ranging from resistance/avoidance to enthusiastic commitment.	Integrative, multi-level model—linking organizational climate (shared perceptions of support/reward/expectations) and innovation-values fit (alignment with user/group/organizational values) to explain implementation outcomes Outcomes range from resistance, avoidance, and compliance to enthusiastic, committed use Effectiveness depends on both strong implementation climate and good values-fit Explains divergent implementation outcomes across groups and organizations Discusses strategies for managing, researching, and improving implementation (e.g., training, incentives, leadership, participatory decision making, value alignment)
GENERAL INNOVATION THEORY	Typology Detail: Policy Innovation	Goyal & Pattyn (2024) [15]	Policy Innovation Framework	POLICY INNOVATION: Radical or transformative changes in public policy—including changes in framing, instruments, processes, practices, or structures. Distinguishes innovation from routine policy change; emphasizes comparative analysis to understand drivers, enabling/failure conditions, impacts, and lessons across contexts. Broader conceptualization to cover programmatic, process, political, democratic, governance, and public sector innovation. Highlights innovation as ongoing practice (not one-time adoption).	Responds to global challenges (climate, inequality, tech disruption) Comparative analysis identifies transferable lessons, enabling/failure conditions Broad conceptualization covers programmatic, process, practice, structure, democratic, governance innovation Interaction of institutions, power, knowledge multiple process theories used (ACF, MSF, others) Methodological innovation—process tracing, QCA, topic modelling, NLP, mixed/multi-methods, large-N studies New research directions: theory-building, digital/tech-policy co-evolution, global south, under-explored domains, negative impacts (dark side)
GENERAL INNOVATION THEORY	Typology Detail: Policy Innovation	Glor (1997) [62]	Policy Innovation Framework	POLICY INNOVATION: Defined as the conception, early adoption and implementation of significant new services, ideas or ways of doing things as government policy in order to improve or reform services, ideas and ways of doing things. Innovation, for Glor, requires “newness” (i.e., never before done in North America in this way, or pilot tested but not previously adopted as government policy) and the element of being an “early adopter” among governments. Minor changes alone do not qualify as innovation; rather, innovations must be significant and substantively improve or reform public sector processes or outcomes.	Public sector innovation is cyclic: it requires feedback, ongoing development, and fine-tuning to create a culture of excellence and adaptability. Success factors include political support, early adoption, and alignment with political agendas; failure or weak adoption may result when media, interest groups, or public support are lacking. Emphasizes need for governmental flexibility, fit with prevailing political platforms, and responsiveness to public attitudes for continual or repeated innovation.

**Table 2:** Academic institution, professional organization, and official international guideline concept descriptions with key characteristics

Source Organization	Description of Innovation	Key Characteristics / Attributes
OECD/Eurostat [63]	A new or improved product or process (or combination thereof) that differs significantly from previous products/processes and is made available to users or put into use.	Novelty within entity/organization, implementation essential (not just invention), value creation, applies to product and process innovation, cross-sectoral inclusivity, robust for measurement/comparison
UK Government [64]	The successful exploitation of new ideas or those adopted from other sectors/organizations.	Emphasizes success and implementation; includes technology transfer/adaptation, focuses on organizational context and competitive advantage
Australian National Audit Office [65]	Creation and application of good ideas within organizational or public sector context.	Continuous/dynamic process; value is central; stress on improvement and relevance to public sector clients/key partners
Confederation of British Industry [66]	Service innovation is excellence achieved via new or improved services or practices.	Focus on improvement, quality, organizational and service contexts; outcome-driven
Economic & Social Research Council, UK [67]	Successful introduction of new services, products, processes, business models, and ways of working.	Includes business models and organizational forms; success linked to adoption/adaptation, organizational change, process and service innovation
American Nurses Association [1]	Application of creativity or problem solving resulting in widely adopted strategy, product, or service meeting a need in a new/different way; about improvement in quality, cost, or efficiency. Innovation involves developing foresight and futures thinking leadership skills and advancing creative ideas to design innovative twenty-first century healthcare systems through interprofessional collaboration.	Competency: creativity, boundary-spanning, change management, considered risk-taking, failure tolerance; linked to standards and competencies for nursing practice; outcome: improved quality, cost-effectiveness, and efficiency; supported by education and communities of practice, foresight, futures thinking, and interprofessional collaboration
ANCC Magnet Recognition Program (2023) [7]	Innovation is “the introduction of something new or a change in the thought process for doing something or the way of doing something,” with emphasis on putting new ideas into practice or incorporating existing ideas in new ways (Melnyk & Fineout-Overholt (2019); Magnet Glossary). Functionally, Magnet organizations are expected to demonstrate new knowledge, innovations, and improvements – including new models of care, application of evidence, or visible contributions to the science of nursing.	Focus on implementation and transformation; includes both introduction of new things and meaningful changes to established practices; innovations must lead to measurable improvements in patient care, operations, or nursing science; expectation of organizational and system impact; supports and recognizes both the generation and practical application of innovations.
Johnson & Johnson Nursing [68]	Nurse-led, creative problem-solving that uses on-the-ground experience and data to design solutions—including new care models, safety practices, and technologies—to improve patient care and create a healthier nursing environment. This definition emphasizes the application of tools like design thinking, the integration of patient and community needs, and the development of scalable solutions from the bedside upwards to tackle complex healthcare challenges and transform healthcare systems.	Nurse-led and patient-first, problem-solving approach, beyond new products, use of design thinking, focus on scalable solutions, collaborative environment, empowerment and support
Society of Nurse Scientists, Innovators, Entrepreneurs & Leaders [69]	The application of creativity and problem-solving to create widely adopted, sustainable strategies, products, technologies, or services that meet a need in a novel way.	Improving quality, cost-effectiveness, or efficiency, leveraging human-centered design, considering ethical implications, ensuring market viability, and providing benefits for frontline caregivers
University of Michigan School of Nursing [70]	Nursing innovation is simply doing something new or thinking about something in a new way - a new process, a new QI improvement, a new clinical practice, a new program, or an intervention. Something nurses already do naturally at the bedside and the research benchtop.	Natural nursing behavior, bedside innovation, curiosity, transformative impact
World Health Organization [71]	New or improved solutions that can accelerate positive impacts on health, encompassing advancements like policies, practices, systems, products, technologies, services, and delivery methods that enhance efficiency, effectiveness, quality, sustainability, safety, or affordability.	Transformative impact, broad scope, focus on health improvement, key partner-centric. May include innovations in the workforce, technology, policy and systems, or community-driven initiatives.

#### Quality assessment

The Smith & Mörelius (2021) quality assessment criteria were used to appraise each of the scholarly sources in the analysis table, evaluating based on epistemology, pragmatic, linguistic, and logical criteria to generate a total score out of 14 total points [72]. Articles were scored 0 to 14 by assigning two points for criteria met, one for criteria partly met, and zero for criteria not met across the four principles. Following the Smith and Mörelius guidelines, scores of 11 or higher indicate robust conceptual contribution and therefore qualify as high-quality material for principle-based analysis [72]. The quality score assessed for each article is detailed in Supplementary Table 2. All articles included in the final sample scored 11 or above.

## Findings

### Uses of the concepts

*Historical Use and Timeline of Innovation in Nursing*. The concept of nursing innovation has been present since the profession’s inception, but innovations were often unrecognized because they were rooted in caring [73, 74]. For example, embedded in the practices of enslaved and free Black healers, Indigenous women, traditional nurses and lay midwives, and Catholic religious orders that predate Florence Nightingale were innovations that occurred despite exclusion from formal healthcare institutions and impoverished conditions [73, 75–78]. At the time, they were labeled as folk or domestic as opposed to scientific, thus omitting them from formal innovation systems.

From 1930 through the post-World War II period, nurses were systemically marginalized from innovation as physicians consolidated medical authority [79]. Nurses were expected to implement innovations handed down from physicians or other professionals rather than serve as innovators themselves. However, by the 1970s-1990s, innovation in nursing was being referenced more explicitly, such as with the Magnet® recognition program linking innovation with nursing excellence [80]. During this period, innovation was also visible in the rise of nursing informatics, including early work on computerized nursing information systems, nursing data representation, quality measurement, and the 1990s formalization of nursing informatics as a specialty [33, 80, 81]. By the 21st century, nursing innovation had been formally institutionalized by incorporating it into practice standards and formal reports [2, 4, 6, 81]. Nursing innovation councils, centers, and fellowship programs were implemented, with frameworks describing product, process, and system-level innovations. Thus, the concept of innovation initially emerged not as a discrete concept, but rather as a product of iterative, creative practice adaptation, sometimes under duress and outside of established medical infrastructure, and progressive professional identity development. For example, nurses in overcrowded hospitals or in remote or segregated clinics often improvised equipment or reworked care practices when formal infrastructure did not support innovation. Over time, the field has moved beyond technological dominance to encompass a variety of typologies within the nursing scope of influence, in both traditional and non-traditional practice settings.

### Definitions

*Dictionary*. Supplementary Table 3 characterizes the difference between nursing innovation and related concepts using dictionary definitions. The dictionary definitions of *innovation* and related concepts all consistently describe the introduction of something new but they differ in their scope and emphasis. *Creativity* is the imaginative ability to produce, *ideation* is the act of forming ideas, and *innovation* is the application of those ideas into something new. *Invention* marks the concrete origination of something, while *novelty* emphasizes the quality of newness itself without concern for utility or imagination. *Health innovation* and *nursing innovation* as explicitly defined terms were not mentioned in online dictionaries or etymology sources.

*Academic, Government, and Professional Organizations Definitions & Use*. Table 2 details the academic, government, and professional organization definitions. These sources share an emphasis on implementation, improvements, and value creation from new ideas. Nonetheless, comparable to the dictionary definitions, they differ in scope and emphasis. The economic and governmental bodies emphasize competitiveness and successful exploitation of ideas for strategic advantage. They reinforce standardization to allow for comparability and benchmarking across nations or institutions. In contrast, the nursing and health-focused organizations emphasize human-centered design, collaborative problem solving, and future thinking as key competencies in addition to tangible outcomes. For nursing, innovation is defined not only as an outcome but also as a relational and collaborative professional practice that is ethically accountable within complex healthcare systems.

### Differentiation of nursing innovation from

*Healthcare Innovation.* The articles in Table 1 provide insight into the boundaries previously drawn between nursing innovation and healthcare innovation literature. Much of the literature to-date has characterized the concept of nursing innovation as a function of professional identity actualization, while healthcare innovation was presented as a means of system optimization [1, 24, 25, 33, 36, 45]. For example, many of the articles described nursing innovation as operating within a bounded universality, or innovation that is simultaneously constrained by, while universally applicable within, professional boundaries [1, 10, 11, 25, 35, 36, 46]. Nursing innovation emphasized professional identity through the lens of caring imperatives and nursing-specific values. Nurse inventors are described as altruistic practitioners [25] and the purpose of technological creativity was described as a means of expressing caring for patients [45]. Any nurse, anywhere, can engage in caring-driven innovation within the scope of nursing professional practice. This emphasis on professional scope has not been mirrored to the same extent outside of nursing literature about innovation.

In contrast to nursing innovation’s bounded universality, healthcare innovation was consistently described as operating with systemic ubiquity, or innovation that transcends professional boundaries and permeates entire systems [51]. Healthcare innovation was defined more often by commercial implementation and economic optimization, an overt economic framing frequently rejected or minimized within nursing innovation literature that so far has emphasized care improvement over commercial and market success.

However, there was evidence that nursing’s bounded universality may be changing as boundaries are being reconsidered [1, 10, 11, 23, 25]. For example, Beaudet et al. (2023) and Barr et al. (2021) described nursing innovation operating across systems, not bounded, while the increasing number of blended transdisciplinary programs (e.g., nursing/architecture, nursing/engineering, nursing/artificial intelligence (AI)), suggested a growing awareness that traditional boundaries may be limiting and that nurses’ scope of influence and practice are expanding [1, 10, 82].

Finally, both nursing and healthcare innovation literature emphasized the importance of individual-level innovation capacity (e.g., behavioral creativity, innovative behavior, and innovativeness as measurable traits) within systems-level approaches [8, 24, 25, 33, 39, 45, 52, 83]. The article by Hewko (2022) used an empirically rigorous, quantitative approach to measuring the relative contributions of individual-level, job-specific, and organizational factors to healthcare innovation output [52]. Specifically, using hierarchical regression, Hewko found that individual factors were most predictive, with role innovation (0.43 coefficient), conscientiousness (0.17), and voluntary membership (0.11) the strongest predictors [52]. These personal traits and behaviors were independent of professional context. Organizational and job-specific factors were significant contributors to the model, but their relative contribution was minimal (i.e., 4% of the variance each). While nursing articles consistently emphasized the importance of individual characteristics, many described them conceptually, within organizational frameworks, or linked to nursing’s professional identity [1, 8, 24, 33, 36, 45, 83].

*General Innovation Theories*. Similar to health innovation, general innovation theory was described as operating through an economics-first imperative while nursing innovation was characterized as operating through predominantly moral and caring imperatives (Table 1). The “altruistic practitioner” nurse inventor develops innovations “for the betterment of patients, professionals, or healthcare institutions” [45]. Success was primarily validated through care improvement rather than economic metrics, although most articles mentioned economic imperatives as an element of innovation. For example, 14 of the 20 nursing articles included a dual imperative, including both overt or implied moral, professional obligation framing and at least a mention of economic pressures, efficiency, or cost imperatives [1, 8, 10, 11, 24, 25, 32, 33, 35, 36, 41, 42, 45, 84]. Two additional studies [40, 83] described a moral imperative without mention of economic imperatives.

In contrast, general innovation theory emphasized competitive advantage and market success to a greater extent than nursing. Social and environmental objectives were incorporated into general innovation frameworks like the Organization for Economic Cooperation and Development (OECD, 2018) [63], though values may be secondary considerations or affect implementation rather than generation. The literature described a primary purpose of general innovation as excelling at market competition and strategic advantage.

### Specification of the defining attributes

Defining attributes are those essential, recurring characteristics that distinguish and clarify the concept of interest [29]. Five defining attributes of nursing innovation were identified from the literature and are detailed in Table 3. The five attributes include nursing agency, contextual novelty, purposeful orientation, implementation, and value creation. These five definitional attributes apply across the novice-to-expert continuum, while the stage of the work (i.e., in development vs. fully implemented) reflects progression along the innovation pipeline rather than the nurse’s individual expertise level. To avoid losing sight of early-stage innovation while preserving definitional rigor and not over-labeling, projects should be labeled as “nursing innovation in development” or “emerging nursing innovation” if they meet nursing agency, contextual novelty, purposeful orientation, and at least pre-implementation (prototype or simulation with intended users) and evidence of value creation.

**Table 3:** Five essential attributes of nursing innovation

Attribute	Definition	Inclusion Test	Exclude If
1. Nursing Agency	Conceived by, led or co-led by nurses	Documentation of nurses as inventor, PI or Co-PI, or accountable decision-maker. Interdisciplinary work documents nurse in a leadership role with signoff on key decisions throughout the cycle.	Nurses serve only as end users, testers, recruiters, or collectors without documented design or implementation authority. Consulted or champion roles with no traceable decision rights do not qualify. Vendor or other-discipline-led work where nurse input is advisory but not incorporated into the final design is not counted.
2. Contextual Novelty	New to the unit of adoption or meaningfully improved from current practice	Clearly described change is substantively new to this context, not routine optimization.	Cosmetic tweaks, routine compliance steps, or rebranding or renewing existing work; standardization without substantive change; “best practice adoption” transplanted without adaptation.
3. Purposeful Orientation	Starts from an explicitly defined problem or unmet need	Problem statement is specific and is situated within nursing’s broad scope of influence	Vague aspiration without a concrete use case; new technology without a defined problem within nursing’s broad scope of practice and influence
4. Implementation	Moves beyond idea generation into use with intended users, with evidence of adoption activities (Stage-dependent)	Documented piloting or uptake by intended users Early-stage – recognize low-fidelity prototyping, simulation, or usability testing with intended users, but label as “pre-implementation” or “pilot-implementation.”	Concepts on paper, proposals, or simulations alone do not qualify
5. Value Creation	Shows progress toward better outcomes or processes that meet a need in a new and different way	At least one credible signal of benefit is documented with consideration for the scope and stage of the work. Early stage – allow provisional value signals (e.g., problem-solution fit, user willingness to adopt, qualitative reports of perceived usefulness), but label as “anticipated value creation.” Realized innovations - one or more robust indicator (clinical, experiential, process, financial, or equity-related outcome) even if preliminary	No plausible value signal; harms outweigh benefits; only satisfaction anecdotes

First, *nursing agency* requires that the work be conceived, led, or co-led by a nurse, with documentation of nurses as inventors, principal or co-principal investigators, leaders, or accountable decision-makers. The word ‘agency’ was chosen as an empowering concept, emphasizing nursing’s capacity to initiate, influence, and direct change rather than imposing limits or boundaries on nursing’s innovation potential. Projects where nurses are only end users, recruiters, or advisors without decision rights are excluded.

Second, *contextual novelty* must be introduced, as the element of newness was present in every definition in this analysis for both nursing and general innovation theories. This means that the change is substantively new to the adopting context, even if not novel elsewhere. This contextual novelty component is aligned with Magnet® standards of nursing excellence [7], and it values the innovativeness required for successful adoption even if not first-in-world inventions. The element of novelty is not met for cosmetic or minor adjustments, compliance steps, or transplanted best practices without meaningful, substantial change.

Third, the work must show a *purposeful orientation*, meaning it begins from a defined problem, unmet need, or opportunity within nursing’s broad scope of influence. These purposes may be defined broadly (e.g., functional, cultural, expressive, or relational) and responsive to diverse, traditional and non-traditional nursing settings, but they still underlie the intentional creation or adoption of something new that is meaningful in context and aligned with the profession’s values, standards, and ethics. Vague aspirations or technology introductions, novelty for its own sake, and innovations that may be harmful or unsafe should not be considered nursing innovations.

Fourth, the innovation must be *implemented*, moving beyond idea generation to documented piloting or uptake by intended users, as opposed to remaining only at the proposal or simulation stage. Within nursing, this criterion addresses the long-standing gap between idea generation and formal adoption, a gap that has historically rendered many nursing innovations invisible or unmeasured. Requiring implementation provides the empirical hinge that distinguishes innovation from routine adaptation, supports attribution to nursing agency, and enables the concept to function as a discrete and testable construct rather than a descriptive aspiration.

Finally, the innovation must *create value* for the intended user, demonstrated by at least one credible signal of benefit in outcomes or processes. An innovation can be intentionally introduced (i.e., purposeful orientation) for aesthetics, symbolism, or cultural meaning without immediately measurable value, but for it to be fully recognized as a nursing innovation, it should eventually create or contribute value for the intended user or unit of adoption. Work that lacks plausible value or change without meaningful impact, or where harms outweigh benefits, fails to meet the criteria for an innovation.

*Typologies of Innovation.* Table 4 details different innovation typologies in nursing, each with varying levels of maturity for the overall profession. As nursing innovation capacity expands, these typologies help to highlight for nurses where their innovation efforts can build on established strengths (i.e., product, process, and organizational innovations) while strategically advancing emerging frontiers in education, policy, marketing, and economic innovation to shape the profession’s future.

**Table 4:** Types of innovation within nursing scope of influence

Innovation Type	Description or Examples in Nursing Innovation	Source(s)
**WHAT NURSES INNOVATE**		
Product Innovation – prototyping, new materials, digital features	Nurse–engineer prototyping of devices and digital tools; nurse-involved prototyping of clinical aids; technology and data innovations; patient care innovations	[43, 45]
Process Innovation – workflow redesign, Lean/Six Sigma, IT adoption	Lean Six Sigma & Plan-Do-Study-Act applied in nursing units; nurse-centered electronic health record/vital sign display redesign, models of care delivery, research and translational methods, internal and team communications and collaborations	[41, 43, 83]
Organizational Innovation – new structures, governance, collaboration models	Nursing innovation centers; shared governance councils; culture-of-innovation initiatives (Magnet exemplars, ANA Innovation Engine)	[1, 4, 8, 10]
Education Innovation – teaching and training redesign	Simulation; hybrid/flipped teaching models; virtual reality/augmented reality for education; NLN Nursing Edge Unscripted podcast, clinical judgement measurement frameworks, AI use in education	[85–87]
Policy Innovation – advocacy, governance, standards	Nurses serving in elected office or governance roles; Climate change & sustainability policies in nursing practice; global health advocacy via united nations delegates; integration of nursing innovation & practice principles into regulatory standards	[15, 21, 62]
Economic and Financial Innovation—value models and financing	Nurse-led venture capital firms (e.g., Nurse Capital); Nursing Human Capital Value Model	[22]
Marketing & Communications Innovation – branding, promotion, new channels, content creation	Sparse in nursing literature. Social media content creators or influencers; Innovation in marketing school programs	[9, 13, 14, 16, 17, 43]
Service Innovation	Care delivery models, nursing role innovations, space nursing, patient experience improvements, telehealth	[18, 19, 32, 42, 46]
**HOW NURSES INNOVATE**		
Design Thinking/Human Centered Design	Structured methodology that centers the needs, experiences, and input of end users (patients, nurses, families) throughout the innovation process, often using empathy-mapping, iterative prototyping, and user feedback. Co-design is frequently utilized as a key component.	[1, 10, 39, 40, 68, 69, 86]
Co-Creation	Overarching process where multiple end users or key partners actively collaborate at every stage of problem-solving, emphasizing mutual contribution and partnership (e.g., Patients, families, and communities co-developing care pathways; interprofessional teams co-designing workflow improvements; partnering with technology developers and engineers to co-build clinical solutions)	[1, 7, 25, 42, 45, 68]
Implementation Science Frameworks	Structured approaches for integrating, evaluating, and sustaining innovations (e.g., consolidated framework for implementation research; reach, effectiveness, adoption, implementation, and maintenance)	[1, 23, 24]
Frugal Innovation	Developing affordable, practical solutions that do more with less by efficiently using limited resources to address healthcare needs, especially in resource-constrained settings	[1, 44, 65, 71]
Lean Startup	Uses rapid cycles of building, measuring, and learning with minimum viable products and user feedback to quickly test or refine new solutions	[63, 68, 69]
Agile Innovation	Relies on rapid, iterative development (e.g., using short sprints and feedback), empowering cross-functional teams to adapt and refine solutions in real time, rather than following lengthy, linear or rigid project phases typical of traditional or waterfall approaches	[63, 68, 69]
Participatory Action Research	Nurses and key partners or end users as equal partners in identifying problems, co-creating and testing solutions, and iteratively refining practices to impact meaningful change; emphasizes local context and sustainability and continuous mutual learning, empowers non-expert participants	[42, 45, 83]
Systems Thinking Innovation	Uses a holistic approach to identify and address connections or patterns across healthcare systems, so nurses can design solutions that improve outcomes for the whole system	[1, 8, 40, 43, 63, 68, 71]
Bottom-Up Innovation	New grassroots ideas, solutions, or improvements that originate from frontline nurses, clinicians, or patients rather than leadership, empowering those closest to the challenges to create meaningful change	[8, 25, 40]
Theory U or Presencing Methodology	Guides teams through deep observation and listening, collective reflection, and prototyping, helping them to sense emerging possibilities and co-create transformative solutions for complex challenges.	None in nursing sources

*Product innovation* (e.g., nurse-engineer prototyping of devices, digital tools) and *process innovation* (e.g., workflow redesign, Lean/Six Sigma, new care models) are well-established and widely practiced in clinical settings [43]. *Organizational innovation* is also mature, evident in nursing innovation centers, Magnet® exemplars, and governance models that embed innovation into culture. *Education innovation* is emerging rapidly, fueled by simulation, virtual and augmented reality, AI-driven teaching models, and new frameworks for clinical judgment [82, 88, 89]. *Policy innovation* is gaining visibility, particularly in climate action, global health advocacy, and regulatory integration of nursing innovation principles. *Economic and financial innovation* is still in early stages, but recent advances—such as nurse-led venture capital firms and human capital valuation models—suggest growing recognition of nursing’s economic impact. In contrast, *marketing and communications* innovation is still emerging in nursing, with limited published work about branding, communication, or social media content creation and strategy. *Service innovations* such as care delivery models, nursing role innovations, and patient experience improvements are well-embedded in nursing innovation discourse but are rarely labeled explicitly as service innovations. *Role innovation* is also an important pathway through which nursing innovation materializes, particularly when nurses extend, redesign, or formalize new domains of practice, authority, or accountability.

*Methods of Innovation in Nursing.* Table 4 also details how nurses innovate. The articles in this analysis mentioned *design-thinking/human centered design, co-creation, implementation science frameworks, frugal innovation, lean startup, agile innovation, participatory action research, systems thinking innovation,* and *bottom-up innovation*. The professional and government sources were more comprehensive about methodology. The peer-reviewed nursing articles and texts showed notable gaps in business/startup methodologies like Lean Startup and Agile. The professional organization reports emphasize viability, but there are gaps in market-oriented approaches in the nursing literature. Surprisingly, despite the prevalence of human-centered design and design-thinking frameworks in non-nursing innovation texts and in the professional organizations, a minority of academic nursing articles explicitly mentioned these frameworks. Similarly, even though a cost focus exists in many nursing articles, frugal innovation methodology was also under-referenced in the nursing articles. Finally, Theory U, a systems-change framework that guides groups from problem sensing to collective prototyping, was not present in any nursing focused literature to the best of our knowledge. These findings suggest a gap in what professional bodies are advocating for, what is being used in broader non-nursing innovation applications, and what the academic research literature conveys. Additional innovation theories, including disruptive innovation, democratizing innovation, and jobs-to-be-done theory, offer useful lenses for future refinement, particularly for examining how nurse-led innovations alter access, redistribute problem-solving capacity, or emerge from unmet user needs.

**Model, Borderline, Related, and Contrary Cases** are presented below, with additional examples in Supplementary Table 4. Several of each are proposed to demonstrate traditional and non-traditional nursing roles and settings as well as various typologies of nursing innovation within the cases. The cases were developed through a combination of illustrative real-world examples and constructed scenarios designed to foreground the concept’s defining attributes and limits.

*Model Case (Exemplifies all attributes) - Service Innovation*. A nurse entrepreneur develops a new service delivery model for prenatal education. She created a digital platform that provides culturally tailored prenatal education through TikTok and YouTube channels, designed specifically for underserved adolescent populations that have difficulty accessing prenatal care through the traditional medical systems. The nurse leads the design and implementation, collaborates with youth advisors, pilots the program with target users, and tracks engagement analytics showing improved knowledge and health-seeking behaviors. This case demonstrates nursing agency (nurse-led), novelty (new use of social media for prenatal care in this context), purposeful orientation (addressing inequities in adolescent maternal health), implementation (piloted with end users), and value creation (documented positive outcomes).

*Borderline Case (Ambiguous application of all attributes) – Marketing & Communications Innovation*. A bilingual nurse influencer with a large Instagram following created “NurseWell IG.” The platform included three streams: (1) short, nurse-authored “Myth vs Fact” micro-lessons on hypertension and type 2 diabetes that linked to guideline summaries; (2) paid posts for a supplement brand using company scripts she could not edit; and (3) personal reflections on nursing life. She designed the editorial calendar, a Spanish track, and a link hub to vetted resources. Linked local federally qualified health centers report modest upticks in clicks to screening sign-ups during her campaigns. Value creation was evident in the large following, but sponsored posts contributed to most of the follower growth and engagement. The platform demonstrates nursing agency in that it was created by the nurse, but the larger contribution of scripted health content from sponsors undermines the nursing agency, therefore this case is borderline. The platform provides a novel solution in nurse-led Spanish micro-lessons at scale with a structured link hub, purposeful orientation (addresses health literacy and increase health screenings), is implemented at scale, and value creation (high follower count, high engagement with posts, increased health screening clicks).

*Related Case (Shares similarities but lacks one defining attribute) – Policy Innovation.* A nurse elected to a state legislature co-sponsors a bill expanding Medicaid reimbursement for telehealth nursing visits. While the initiative advances access and aligns with nursing values, the nurse is not the sole driver of the legislation, and the innovation reflects an adaptation of existing telehealth reimbursement models rather than a substantively novel solution. The case demonstrates nursing agency and purposeful orientation, and it results in policy-level value creation, but its limited contextual novelty and shared authorship position it as a related rather than model case of nursing innovation.

*Contrary Case (Clearly not nursing innovation) – Product/Technology Innovation.* A technology company designs a remote patient-monitoring device and hires nurses solely to test the product and provide user feedback. The project is novel, implemented, and creates value for patients, but nurses are not decision-makers or inventors, serving only as end users. This absence of nursing agency disqualifies the work as a nursing innovation, aligning instead with general healthcare innovation.

**Antecedents and Consequences** – Table 5 summarizes antecedents and consequences retrieved from this sample of articles. Within the literature, we found individual, team, organizational/contextual, professional, environmental and external level, and innovation-specific antecedents and consequences.

**Table 5:** Individual, organizational, professional, and system level antecedents and consequences extracted from the articles in the final sample

Level	Antecedents	Consequences
Individual/Behavioral	• Age, tenure, certification, education level • Clinical expertise and professional knowledge • Innovation competency and risk tolerance • Interpersonal and teamwork skills, leadership power • Motivation to improve care and outcomes • Perfectionism, flexibility, tenacity and determination, self-confidence, conscientiousness, ambition, analytical thinking • Problem-solving and creative abilities, visionary mindset • Psychological empowerment • Tolerance of ambiguity, intellectual ability, values, learning style	• Better capacity for innovation implementation and routinization • Career advancement and leadership opportunities • Enhanced job satisfaction and professional pride • Enhanced knowledge and skill development • Enhanced professional competency and expertise • Enhanced research ability scores • Improved confidence and self-esteem • Improved innovation ability scores • Increased motivation and engagement in work • Increased professional recognition and credibility • Reduced job burnout and stress levels • Strengthened creative thinking and problem solving
Team and Group Level	• Clear communication, cohesion, precise purpose or vision, mix of skills and experiences, trust and confidence, accountability, transparent leadership • Homophily (similar backgrounds) • Professional autonomy and independence • Social networks and network structure	• Better capability to adapt and respond to challenges • Better integration of different processes and perspectives • Enhanced communication across structural boundaries • Enhanced knowledge sharing within organizations • Improved absorptive capacity for new knowledge • Improved team effectiveness and collaboration • Increased collective innovation capacity • Strengthened innovation climate and culture • Stronger team synergy and interprofessional partnerships
Organizational/Contextual	• Access to equipment and facilities • Access to learning opportunities, rewards, and incentives • Creative Climate for innovation, Supportive culture • Financial resources, time allocation • Formalization levels • Leadership commitment, style, and vision, attitudes towards change, and resource availability • Organizational size, maturity, functional differentiation, decentralization • Procedural justice • Psychological safety for experimentation • Recognition and reward systems • Slack resources, specialization, administrative intensity • Training and development programs	• Better alignment between innovation and strategic objectives • Better profit margins on innovative solutions • Better return on investment in innovation activities • Competitive advantage and market differentiation • Cost savings and operational optimization, better resource utilization and waste reduction • Enhanced organizational adaptability and resilience • Enhanced organizational culture and engagement • Improved capacity for continuous improvement • Improved economic sustainability of organizations • Improved financial performance and revenue generation • Improved patient outcomes and safety indicators • Improved quality metrics and performance indicators • Increased market share and competitive positioning • Increased organizational learning and knowledge management • Strengthened innovation management systems
Professional	• Access to evidence and research resources • Boundary spanners • Communication channels and networks • Continuing education and opportunities • Educational preparation and professional networks • Expert promoters and power promoters • Opinion leaders and champions • Professional autonomy and decision-making authority	• Advancement of professional knowledge base • Better clinical effectiveness and evidence-based practice • Better health promotion and disease prevention • Enhanced access to healthcare services • Enhanced patient satisfaction and care experiences • Improved care coordination and continuity • Improved care delivery for underserved populations • Influence on nursing education and practice standards • Policy development and regulatory changes • Professional recognition and credibility enhancement • Reduced adverse events and complications • Reduced health disparities
Environmental and External	• Academic-practice partnerships • Assessment of implications • Capacity to evaluate innovation • Dedicated time and resources • Environmental uncertainty and dynamics • Funding mechanisms and investment sources • Healthcare policies supporting innovation • Interorganizational networks and collaboration • Market competition and customer demands • Political directives and policy support • Power balances between supporters and opponents • Quality measurement infrastructure • Sociopolitical climate • Tension for change, innovation-system fit	• Better translation of research into practice • Economic impact and value creation • Enhanced evidence base for clinical practice • Generation of new knowledge and best practices • Healthcare transformation across multiple domains • Improved dissemination of innovations across organizations • Knowledge advancement and evidence base expansion • Population health improvement and equity advancement • Strengthened infrastructure and capacity • Strengthened scientific foundation for nursing practice
Innovation-Specific	• Innovation-values fit • Perceived usefulness and ease of use • Relative advantage, compatibility, complexity, trialability, observability • Risk level and uncertainty	• Better adaptation and reinvention of innovations for local contexts • Better innovation success rates and outcomes • Development of new products, services, processes • Enhanced innovation pipeline and portfolio management • Enhanced sustainability of innovative practices • Improved scaling and spread of successful innovations • Increased patent applications and intellectual property development • Successful implementation and routinization of innovations

At the *individual level*, antecedents cluster around creativity, problem-solving, and psychological empowerment. The literature at times conflates these antecedents with consequences, treating some traits as both inputs and outcomes. For example, creativity was described as both an individual trait and a behavior, both of which are consistently linked to or used interchangeably with innovativeness in the nursing literature. This contributes to conceptual vagueness within the existing frameworks, supporting the need for a refinement. This finding also suggested a feedback loop where innovating not only requires but also reinforces individual competencies, a nuance underexplored in existing work. If this feedback loop exists, it implies that investing in nursing innovation capabilities can have exponential returns by building the strengths that perpetuate innovation (i.e., innovative behaviors, individual traits), leading to a self-sustaining cycle of growth. While previous work has described innovation as an iterative or cyclical process, our findings uniquely suggest the existence of a compounding effect on workforce development and system capacity, even when the innovations themselves are unsuccessful.

At the *team level*, antecedents such as cohesion, trust, and diverse skill sets lead to consequences like enhanced absorptive capacity and collective innovation. Yet the literature rarely interrogated how homophily, or the formation of innovation teams based on similarity in background or values, may both enable and constrain innovation [85]. In nursing, homophily might manifest as either clustering only with other nurses or deliberate interprofessional engagement. When overextended, a commitment to interprofessional engagement may lead to dilution of nursing’s visible contributions. The dual role of similarity in facilitating trust but potentially limiting novel perspectives warrants further research in nursing contexts, with and without interprofessional engagement.

At the *organizational level*, nursing innovation literature consistently described the need for supportive environments for creative experimentation and states that while a majority are willing to innovate, few feel they can always do so in daily work [25, 36, 39]. Nearly all nursing innovation frameworks linked innovation success to organizational infrastructure, culture, and leadership. Here, a gap inherent in previous work is the assumption that all supportive climates translate linearly into financial or quality gains. For example, few studies examined failed or stalled organizational innovations, leaving an incomplete understanding of negative or neutral consequences associated with formal organizational infrastructure. In addition, the allied health work by Hewko (2022) appears to refuse this assumption, because they found that the relative contribution of the organization to innovation outputs was minimal (4%), and that an individual’s innovative behaviors were predictive of innovation output without being context dependent [52]. Comparable work has not yet been reported in nursing, to the best of our knowledge.

The emphasis on organizational infrastructure was a necessary and catalyzing element of integrating innovation into the mainstream of nursing practice. However, now that nursing has reached the competent level of Benner’s ladder [5], there is a need for a more nuanced and rigorous investigation of the organizations role as we advance towards proficient and expert. First, existing organization-dependent frameworks do not account for our profession’s history when innovation occurred largely outside of formal institutions and under low-resource conditions. Next, current frameworks also did not account for innovation in non-traditional roles and settings, such as social media content creators or nurses impacting policy innovation as elected officials or in global diplomacy positions. Finally, as more nurses approach the proficient and expert stages on the novice-expert continuum, they may become less dependent upon an organization’s culture and infrastructure to innovate. New frameworks should account for nursing innovation both within and outside of a supportive, fully-resourced organizational context, while also delineating the relationships between the innovator and the organization across the novice-expert continuum.

At the *professional and system levels*, antecedents such as access to research, boundary spanners, and academic-practice partnerships linked consequences like policy influence and population health improvement. However, these levels are often treated aspirationally [25]. The mechanisms through which professional and policy antecedents cascade into measurable outcomes remain thinly described in current nursing literature. Future research is needed to develop robust evidence pathways or empirical evidence of the causal effects at policy and population levels.

*Environmental and external antecedents* reflect forces outside the immediate organization that shape whether nursing innovations can gain traction, including academic–practice partnerships, funding mechanisms, policy directives, market pressures, interorganizational networks, and broader sociopolitical conditions. These factors determine the permeability of the innovation environment. The *consequences* appear at population and system levels where the visibility and strength of the evidence may shift outcomes. At this scale, the environment can amplify nursing or impede it, altering how fully it contributes to improved care and a more equitable health system.

Finally, *innovation-specific* antecedents (e.g., fit, relative advantage, observability) align with classic innovation-diffusion theory [48, 55], but nursing innovation adds a unique emphasis on values fit and safety. This moral dimension reframes success not only as scale or profitability but as fidelity to professional ethics, a contribution which is less visible in the broader innovation literature. The perceived usefulness and ease of use of an intervention is linked to increased adoption rates, but usefulness is often defined by safety, care processes, or wellbeing of staff and patients over market share or revenue. Finally, the nursing literature uniquely weights risk in terms of reputational and clinical risks, not only in terms of the business or financial risks which are centered in the broader innovation literature. And while any healthcare innovations will consider safety and risk, nursing elevates this aspect to core values that influence development and adoption.

### Empirical referents

Empirical referents in the Walker and Avant (2019) concept analysis process are the observable, measurable phenomena that demonstrate the occurrence of the concept in practice [29]. We extracted 124 unique constructs derived from 147 original empirical referents, detailed in Supplementary Table 5 (i.e., nursing agency (29 constructs), contextual novelty (26), purposeful orientation (18), implementation (27), and value creation (24)). Each extracted empirical referent construct was listed in the table with one or more example operational definition(s) and coded as addressing either moral imperatives, economic imperatives, or both. We found that most (90%, 112 constructs) addressed both moral and economic imperatives. Eight constructs (7%) addressed the moral imperative only, and four constructs (3%) were economic only.

For *nursing agency*, empirical referents demonstrated nurses as autonomous professionals who exercise creative thinking, emotional engagement, collaborative leadership, and professional inventiveness to initiate and champion solutions. Examples include nurses listed on patents or intellectual property filings as inventors; publication, protocols, or policy authorship; company ownership linking to the innovation; curriculum documents or syllabi; or Magnet® exemplars where nurses are documented as leading the project or accountable decision-makers throughout.

For *contextual novelty*, empirical referents reflect a wide spectrum of nursing innovations ranging from tangible technologies and devices (e.g., mattresses, positioning equipment, and global positioning systems) to intangible care delivery models, educational programs, and organizational structures.

Empirical referents of *purposeful orientation* show innovation as driven by nurses’ ability to identify opportunities, assess complex problems, and apply systematic approaches across nursing’s broad scope of influence. Evidence may include a clearly articulated problem statement in project charters, institutional review board protocols, or quality improvement proposals that positions the innovation within nursing’s broad scope of influence.

The *implementation* empirical referents describe structured processes through which innovative ideas progress from conception through prototyping, testing, dissemination, and sustainable integration into regular nursing practice and organizational workflows. Sources of evidence may include proof of piloting or uptake such as pilot project reports, adoption in the clinical workflows, training modules delivered to staff, or integration into curricula.

Finally, the empirical referents of *value creation* capture diverse outcomes including enhanced patient safety and outcomes, organizational financial performance, nurse job satisfaction and productivity, community health improvements, and transformation of healthcare. Given the authority on value creation was the intended user and key partners, the empirical referents should meticulously document their feedback.

Gaps exist in the literature about empirical referents for nurses working in non-traditional settings, roles, or under-resourced or independent environments. Notable gaps in the nursing literature exist for content creators or digital innovation, policy innovation, venture capitalists or financial innovation, under-resourced or remote settings, and independent entrepreneurial practice. Empirical referents for influencers and content creators may include social media engagement metrics or follower impact on health behavior change, digital content creation productivity, health misinformation correction effectiveness measures, cross-platform influence and reach indicators, and patient education content consumption and behavior modification outcomes [14]. For policy innovation, missing from the literature is legislative proposal passage rates by nurse legislators, policy impact measurements on population health outcomes, political astuteness scores or voting behavior patterns for nurse constituents, or coalition building and bipartisan collaboration success rates. For venture capitalist or financial innovation, empirical referents missing from the literature include investment portfolio performance metrics specific to nurse-led startups, mentorship effectiveness measures for nurse entrepreneurship, return on investment comparisons between nurse-led vs other healthcare startups, and market validation techniques for nursing-specific innovations. Related to global or international settings, missing from this literature are cross-cultural innovation adaptation success rates, global application of frugal innovation outcomes, international collaboration and knowledge transfer metrics, global health impact measures from nursing innovations, and sustainability indicators for diverse economic contexts.

Empirical referents for under-resourced or remote settings that were absent from this sample of articles include resource-constraint innovation measures (e.g., innovations per dollar invested), mobile health solution effectiveness in areas with limited infrastructure, or sustainable innovation implementation without organizational support.

For innovation independent of organizations or entrepreneurial practice, empirical referents missing from the literature include solo practice innovation development and scaling measures, client outcome improvements in nurse-owned practices, business sustainability measures for nurse entrepreneurs, innovation diffusion rates outside traditional healthcare organizations, or professional network leveraging effectiveness for independent innovators.

## Discussion

After appraising the literature with an integrated scoping review and concept analysis methodology, we identified five essential elements of nursing innovation that clarify but do not confine the concept: nursing agency, contextual novelty, purposeful orientation, implementation, and value creation. Nursing innovation was defined as the exercise of nursing agency to design, implement, and evaluate contextually novel and purposefully oriented approaches that create value and address challenges across the profession’s broad and expanding domains of influence.

Unlike previous frameworks that positioned nursing innovation as dependent upon organizational endorsement and institutional support, this analysis recognized innovation across the full spectrum of nursing practice. The term ‘agency’ was selected to empower, as it invites nurses to expand their scope and assert new directions while maintaining professional accountability without limits implied by historic definitions. Novelty underscores that contextual newness is sufficient without requiring first-in-world. Purposeful orientation grounds innovation in nursing’s professional values and ethics without precluding engagement with the efficiency, sustainability, or economic imperatives emphasized in general innovation theories. Implementation requires that ideas move beyond conceptualization into use, while value creation ensures meaningful impact to the intended user or unit of adoption. This framing positions nurses to embrace the pragmatic and economic realities of healthcare without abandoning the professions values, offering a definition that expands possibilities rather than imposes limits.

Although this analysis treats nursing innovation as a distinct construct, the intent is not to separate nurse-led work from broader healthcare innovation. The distinction is useful only if it improves attribution, measurement, and translation. Without clear attribution to nursing agency, nurse-led contributions may remain invisible or be absorbed into organizational or medical innovation. However, if the term is used too narrowly, it may also limit diffusion beyond nursing audiences. A more useful framing is to define nursing innovation by the presence of accountable nursing agency while evaluating its value at the level where the innovation operates, including patients, organizations, markets, policy, or health systems.

Historically, nursing innovations were underrecognized, often subsumed under medical or organizational achievements. This marginalization limits diffusion and visibility. Current literature seeks to reclaim nursing’s distinct contributions, highlighting its expanding scope into policy, finance, marketing, and global health. Nursing innovation frameworks that incorporate evidence-based practices for advancing equity for historically marginalized groups are needed, as emphasized by numerous frameworks and professional organizations [10, 68]. Formal tracking systems and empirical referents are needed that allow local organizations to benchmark their progress toward resolving the diversity-innovation paradox over time.

This analysis suggests that nursing innovation and general innovation so far have operated as distinct paradigms rather than variations of the same phenomenon. In the literature reviewed, nursing innovation was often framed through a moral or caring-dominant paradigm, whereas general innovation and healthcare innovation more often emphasized economic, market, or system-optimization imperatives. This distinction should not be interpreted as meaning that nursing innovation is inherently altruistic or limited to caring-related interventions. Rather, it reflects a dominant pattern in the literature to date, even as contemporary nursing innovation increasingly includes role, policy, technological, educational, entrepreneurial, and economic forms.

This pattern may explain why market-based healthcare innovation often fails to fully engage nurses and why nurse-led innovations struggle with scaling through commercial mechanisms. These differences create several theoretical gaps. First, general innovation theory does not adequately categorize innovations where the primary driver is moral obligation. Nursing innovations that improve care without commercial potential become theoretically invisible or misclassified as “failed” innovations. The dominant success metrics of market share, profitability, and competitive advantage distort the understanding of caring-driven innovations. Second, the general innovation frameworks like Klein & Sorra’s (1996) “innovation-values fit” and Rogers’ (2003) “relative advantage” assume that economic or efficiency benefits drive adoption, and as such are unable to explain why nurses adopt innovations primarily because they enhance caring capacity, even when these innovations increase workload or complexity [55, 61].

On the other hand, nursing innovation’s traditional link to bounded universality and the moral imperative create systematic limitations, or ‘virtuous constraints,’ limiting impact for both the profession and for individuals. The mismatched metrics render nursing innovation systematically invisible to traditional funding mechanisms, which is one of the most significant barriers to nursing innovation [26]. Without demonstrable return on investment in terms that traditional funding bodies understand, nurse innovators may appear “unfundable.” This occurs because funding bodies operate through economic imperatives that cannot recognize or validate caring-driven innovation. Further, nurse altruism paradoxically penalizes nurses in mainstream funding competitions, where self-interest and profit motivation signal viability to investors and grant committees. The rise of nurse-led venture capital firms and national nursing innovation programs (i.e., Johnson and Johnson’s and ANA’s innovation engines [1, 68]) may help to further shift this dynamic to articulate the value of moral innovation paradigms and create more sustainability options for nurse-led ventures.

The caring imperative also creates a care-first priority conflict. As nurses and organizations prioritize patient care, innovation becomes a secondary concern that becomes chronically deferred. Nurse-led ideas are at risk of being passed on to others to patent, implement, and sustain. As such, the care-first imperative that drives nursing innovation simultaneously prevents its development. This also puts nurses at risk of moral exhaustion because it demands continuous moral engagement.

Further, the professional boundary limitation of previous nursing innovation definitions may limit nurses’ impact, even as it creates deep value within those boundaries. The dependence upon professional networks for spread and adoption means that innovations may require translation to spread beyond nursing to others who operate through different imperatives. In addition, the extracted articles in this analysis tie innovation to professional identity. This may create innovations that are aligned with nursing values but may appear irrelevant or non-essential to others. The anti-commercial orientation of nursing literature to-date may perpetuate the creation of improvements that lack sustainable resource generation that occurs with general innovation theory. Our profession should embrace a more balanced approach to both the moral and economic imperatives for innovation.

For typologies and methodologies, nursing demonstrates sophisticated application of participatory action research and systems thinking innovation yet shows limited adoption of lean startup and agile methods that dominate current innovation ecosystems. This pattern suggests that nursing may need to integrate entrepreneurial approaches while preserving its collaborative innovation strengths. The absence of Theory U methodology combined with strong bottom-up innovation documentation to-date suggests a preference for grassroots problem-solving over transformational leadership frameworks. This approach may create advantages in patient-centered innovation but may limit access to tools for large-scale systemic change. Finally, nursing excels at service innovations but rarely labels them explicitly as such, creating an opportunity to lead healthcare service innovation discourse while learning from service design methodologies in other industries by formally incorporating it into curriculum and publications.

Empirical referents found in the literature are currently skewed toward nurses working in traditional healthcare organizations (i.e., hospitals, health systems, academic institutions) with established or intent to establish innovation infrastructure, leaving significant measurement gaps for the growing population of nurses working independently, in non-traditional roles, in ambulatory areas or in highly resource-constrained environments.

Though this review identified antecedents and consequences of nursing innovation that span individual, team, organizational, professional, and system levels, several underexplored gaps were noted. The frameworks do not address varying levels of competencies across the novice-to-expert continuum; antecedents and consequences are treated as uniform, without accounting for how they may shift as nurses progress from novice to expert. Future work should also disentangle how antecedents function differently as traits, behaviors, or contextual conditions, and test whether they act as reinforcing loops rather than linear inputs. Even though the models acknowledge that the process of innovation is iterative, the antecedents and consequences are treated as discrete and one-directional when in practice they may amplify each other. Finally, there is a need to examine antecedents and consequences beyond well-resourced formal organizations, including non-traditional roles and settings, while clarifying how professional and policy antecedents translate into measurable outcomes.

Our proposed model, related, borderline, and contrary cases illustrate how the defining attributes distinguish nursing innovation from adjacent activities. Traditional cases highlight how incremental changes or borrowed tools may fall into related or borderline territory, while non-traditional examples in policy, marketing, and economic domains demonstrate the expanding boundaries of nursing innovation and the importance of maintaining agency, contextual novelty, purposeful orientation, implementation, and value creation as the criteria for inclusion.

### Limitations

Although scholarly peer reviewed sources are a gold standard for concept analyses and scoping reviews, the nature of innovation in practice does not lend itself to extensive and current scholarly publications (e.g., the need to abstain from public disclosure until the patenting process is complete, its function for competitive advantage within organizations). As a result, this analysis was limited by what was accessible in the literature. Most extracted sources focused on hospitals and academic settings, underrepresenting nurses who work in independent practice, policy, entrepreneurship, or global health. In addition, this literature is United States and Europe-centric, with limited perspectives found from low- and middle-income countries where innovation often arises under constraint.

## Conclusions

This analysis advances the concept of nursing innovation by clarifying its essential attributes and broadening its scope beyond traditional organizational and clinical settings into non-traditional or under-resourced nursing roles and settings. By introducing nursing agency, contextual novelty, purposeful orientation, implementation, and value creation as defining features, it establishes boundaries that distinguish nursing innovation from general healthcare and management innovation while avoiding limits that constrain its potential. This analysis highlights gaps overlooked in prior work, including conflation of antecedents and consequences, the underexplored role of homophily in nursing innovation teams, and the assumption that supportive climates are uniformly and linearly associated with positive outcomes. Importantly, this paper differentiates nursing innovation from the broader innovation paradigm as shaped by both moral and economic imperatives, offering a framework that acknowledges nurses’ values and ethics in a way intended to empower instead of constraining them. In doing so, it positions nursing innovation as not only visible and measurable but also adaptable across the novice-to-expert continuum and transferable to diverse roles and settings, from bedside care to global policy.

## Electronic supplementary material

Below is the link to the electronic supplementary material.

Supplementary Material 1

Supplementary Material 2

Supplementary Material 3

Supplementary Material 4

Supplementary Material 5

## Data Availability

All data generated or analysed during this study are included in this published article and its supplementary information files.

## Abbreviations

AI: Artificial Intelligence
ANA: American Nurses Association
OECD: Organization for Economic Cooperation and Development
PRISMA: Preferred Reporting Items for Systematic reviews and Meta-Analyses
PVC: Pre-ventricular Contraction
SONSIEL: Society of Nurse Scientists, Innovators, Entrepreneurs & Leaders
