# Supplementary material for: Nursing innovation: a comprehensive definition and framework for implementation
Source: BMC Nurs. 2026 Aug 6;25:1056. doi: 10.1186/s12912-026-05124-7 (PMC13729330; doi:10.1186/s12912-026-05124-7)
Supplement: Supplementary file 1 [file 12912_2026_5124_MOESM5_ESM.pdf]

Defining Attribute	Original Empirical Referent	Theoretical/Conceptual Referent	Examples of Operational Measurement(s)
Nursing Agency	Thinking outside the box, having a visionary mind	Creative cognitive capacity	Torrance Test of Creative Thinking; Creative Problem Solving Scale; Divergent thinking assessments
Nursing Agency	Positive emotional engagement including enthusiasm and caring about patients	Affective commitment to patient care	Emotional engagement scales; Patient advocacy behaviors; Caring behavior inventory
Nursing Agency	Team synergy and ability to collaborate with other professionals	Interprofessional collaboration competence	Interprofessional Collaborative Competency Attainment Scale; Team effectiveness measures
Nursing Agency	Independence and intellectual autonomy in practice	Professional practice autonomy	Dempster Practice Behavior Scale; Nursing Work Index autonomy subscale
Nursing Agency	Opportunity exploration and problem recognition	Innovation opportunity sensing	Opportunity recognition scale; Environmental scanning behavior measures
Nursing Agency	Intentional behavior directed at innovation	Innovation behavioral intention	Theory of Planned Behavior scales; Innovation implementation intention measures
Nursing Agency	Individual who takes initiative and ownership of innovation process	Innovation ownership and leadership	Personal initiative scale; Innovation champion behavior inventory
Nursing Agency	Professional autonomy to act as innovators and leaders	Innovation role empowerment	Structural empowerment scale; Innovation climate inventory
Nursing Agency	Nurses leading and developing solutions to complex problems across healthcare	Solution development leadership	Innovation leadership behavior scale; Problem-solving effectiveness measures
Nursing Agency	Nurse-led solution development and creative problem-solving	Autonomous solution creation	Innovation output measures; Solution quality assessments; Creative problem-solving evaluations
Nursing Agency	Altruistic practitioner with creativity and knowledge to collaborate	Innovation behavior: Altruistic practitioner with creativity ...	Innovation behavior scales; Performance measures; Behavioral assessments
Nursing Agency	Nurse inventor as separate professional entity with novel skills	Innovation behavior: Nurse inventor as separate professional ...	Innovation behavior scales; Performance measures; Behavioral assessments
Nursing Agency	Nurse-innovator role in co-creating nursing technologies	Innovation behavior: Nurse-innovator role in co-creating nurs...	Innovation behavior scales; Performance measures; Behavioral assessments
Nursing Agency	Collaborative process involving nurse-innovators and network collaborators	Innovation behavior: Collaborative process involving nurse-in...	Innovation behavior scales; Performance measures; Behavioral assessments
Nursing Agency	Professional inventiveness in creating new nursing approaches	Innovation behavior: Professional inventiveness in creating n...	Innovation behavior scales; Performance measures; Behavioral assessments
Nursing Agency	Individual nurse or teams taking responsibility for implementing innovation	Innovation behavior: Individual nurse or teams taking respons...	Innovation behavior scales; Performance measures; Behavioral assessments
Nursing Agency	Solution-focused nurses making progress in challenging situations	Innovation behavior: Solution-focused nurses making progress ...	Innovation behavior scales; Performance measures; Behavioral assessments
Nursing Agency	Nurses educated to focus on patient or system needs as first priority	Patient care innovation	Care quality measures; Patient satisfaction; Clinical effectiveness indicators
Nursing Agency	Nurses as knowledge workers who understand needs better than other professions	Innovation behavior: Nurses as knowledge workers who understa...	Innovation behavior scales; Performance measures; Behavioral assessments
Nursing Agency	Nurses role-modeling creative approaches to problem-solving	Innovation behavior: Nurses role-modeling creative approaches...	Innovation behavior scales; Performance measures; Behavioral assessments
Nursing Agency	Leadership competency in encouraging staff innovation	Innovation behavior: Leadership competency in encouraging sta...	Innovation behavior scales; Performance measures; Behavioral assessments
Nursing Agency	Nurses with innovation ability to actively seek and develop new methods	Innovation behavior: Nurses with innovation ability to active...	Innovation behavior scales; Performance measures; Behavioral assessments
Nursing Agency	Innovation leaders facilitating team-based innovation approaches	Innovation behavior: Innovation leaders facilitating team-bas...	Innovation behavior scales; Performance measures; Behavioral assessments
Nursing Agency	Nurses moving into areas traditionally viewed as medical practice	Innovation behavior: Nurses moving into areas traditionally v...	Innovation behavior scales; Performance measures; Behavioral assessments
Nursing Agency	Specialist plus concept extending traditional nursing boundaries	Innovation behavior: Specialist plus concept extending tradit...	Innovation behavior scales; Performance measures; Behavioral assessments
Nursing Agency	Individual employee innovativeness as personal characteristic	Innovation behavior: Individual employee innovativeness as pe...	Innovation behavior scales; Performance measures; Behavioral assessments
Nursing Agency	Personal growth initiative as predictor of innovative behavior	Innovation behavior: Personal growth initiative as predictor ...	Innovation behavior scales; Performance measures; Behavioral assessments
Nursing Agency	Nurse leaders including innovation as core expectation for all nurses	Innovation behavior: Nurse leaders including innovation as co...	Innovation behavior scales; Performance measures; Behavioral assessments
Nursing Agency	Individual nurses as initiators of social and communicative innovation process	Innovation behavior: Individual nurses as initiators of socia...	Innovation behavior scales; Performance measures; Behavioral assessments
Contextual Novelty	53 kinds of nursing technologies created including blankets, mattresses, devices	Technological innovation productivity	Patent applications; Technology transfer metrics; Innovation disclosure counts
Contextual Novelty	Practical inventions involving combining devices or creating new functions	Applied innovation capability	Invention effectiveness measures; Practical application success rates
Contextual Novelty	Intellectual inventions as creative approaches to find unknown solutions	Conceptual innovation capacity	Conceptual novelty assessments; Theoretical innovation measures
Contextual Novelty	Idea generation for novel or adopted solutions to problems	Solution ideation capability	Alternative Uses Task; Idea generation fluency measures; Solution originality ratings
Contextual Novelty	New ideas, products, processes, or procedures new to the unit of adoption	Innovation novelty creation	Innovation novelty scale; New product development metrics
Contextual Novelty	Creative solutions that are original and useful to organization	Creative solution quality	Consensual Assessment Technique; Solution creativity ratings; Usefulness evaluations
Contextual Novelty	New nursing tools, machines, nursing procedures, education techniques	Innovation behavior: New nursing tools, machines, nursing pro...	Innovation behavior scales; Performance measures; Behavioral assessments
Contextual Novelty	Seven-phase process creating new technologies: investigating through monitoring	Innovation behavior: Seven-phase process creating new technol...	Innovation behavior scales; Performance measures; Behavioral assessments
Contextual Novelty	Technologies including clothes, utensils, apparatus, utilities, tools, machines	Innovation behavior: Technologies including clothes, utensils...	Innovation behavior scales; Performance measures; Behavioral assessments
Contextual Novelty	Mobile surgical services bringing operating theater to rural communities	Innovation behavior: Mobile surgical services bringing operat...	Innovation behavior scales; Performance measures; Behavioral assessments
Contextual Novelty	Vollman Prone Positioner for critically ill patients	Patient care innovation	Care quality measures; Patient satisfaction; Clinical effectiveness indicators
Contextual Novelty	Native Health Nursing scheme with greater autonomy for nurses	Innovation behavior: Native Health Nursing scheme with greate...	Innovation behavior scales; Performance measures; Behavioral assessments
Contextual Novelty	Nurse-led clinics providing comprehensive primary healthcare	Patient care innovation	Care quality measures; Patient satisfaction; Clinical effectiveness indicators
Contextual Novelty	New care delivery models and nursing practice innovations	Patient care innovation	Care quality measures; Patient satisfaction; Clinical effectiveness indicators
Contextual Novelty	New services or products developed through innovation process	Innovation behavior: New services or products developed throu...	Innovation behavior scales; Performance measures; Behavioral assessments
Contextual Novelty	Faculty innovation fellowships and accelerator programs	Innovation behavior: Faculty innovation fellowships and accel...	Innovation behavior scales; Performance measures; Behavioral assessments
Contextual Novelty	Innovation centers creating structures to support novel approaches	Innovation behavior: Innovation centers creating structures t...	Innovation behavior scales; Performance measures; Behavioral assessments
Contextual Novelty	Novel partnerships and partnership models for sustainability	Innovation behavior: Novel partnerships and partnership model...	Innovation behavior scales; Performance measures; Behavioral assessments
Contextual Novelty	Drawsheet evolution to bed chuck for patient comfort and efficiency	Patient care innovation	Care quality measures; Patient satisfaction; Clinical effectiveness indicators
Contextual Novelty	Creative use of technology to solve nursing challenges	Innovation behavior: Creative use of technology to solve nurs...	Innovation behavior scales; Performance measures; Behavioral assessments
Contextual Novelty	New methods, new technologies and new tools to promote health	Innovation behavior: New methods, new technologies and new to...	Innovation behavior scales; Performance measures; Behavioral assessments
Contextual Novelty	Innovation products developed through workshop-based learning	Innovation behavior: Innovation products developed through wo...	Innovation behavior scales; Performance measures; Behavioral assessments
Contextual Novelty	Six domains of innovative nursing roles: Skill/Task, Role, Condition, Area, Client Group, Generic	Innovation behavior: Six domains of innovative nursing roles:...	Innovation behavior scales; Performance measures; Behavioral assessments
Contextual Novelty	Innovative roles that are entirely new or significantly modified	Innovation behavior: Innovative roles that are entirely new o...	Innovation behavior scales; Performance measures; Behavioral assessments
Contextual Novelty	New or improved products, services, processes developed by nurses	Innovation behavior: New or improved products, services, proc...	Innovation behavior scales; Performance measures; Behavioral assessments
Contextual Novelty	Step Forward program creating mechanism for idea submission and cultivation	Innovation behavior: Step Forward program creating mechanism ...	Innovation behavior scales; Performance measures; Behavioral assessments
Contextual Novelty	Technical innovations including new products and processes	Innovation behavior: Technical innovations including new prod...	Innovation behavior scales; Performance measures; Behavioral assessments
Contextual Novelty	Administrative innovations involving new organizational structures	Innovation behavior: Administrative innovations involving new...	Innovation behavior scales; Performance measures; Behavioral assessments
Contextual Novelty	GPS technology innovation allowing free walking while maintaining safety	Innovation behavior: GPS technology innovation allowing free ...	Innovation behavior scales; Performance measures; Behavioral assessments
Contextual Novelty	Social innovation practices reconfiguring social relations	Innovation behavior: Social innovation practices reconfigurin...	Innovation behavior scales; Performance measures; Behavioral assessments
Purposeful Orientation	Innovation begins with coworkers identifying new opportunities	Collaborative innovation initiation	Team innovation behavior scale; Collaborative problem identification measures
Purposeful Orientation	Focus on what is possible and what is needed simultaneously	Need-feasibility integration	Needs assessment accuracy; Feasibility evaluation skills; Gap analysis competence
Purposeful Orientation	Innovation essential to solve wicked problems in healthcare	Complex Purposeful Orientation	Wicked problem recognition scale; Complex problem-solving measures; Systems thinking assessments
Purposeful Orientation	Creative problem-solving for patient health problems and nurse challenges	Patient-centered problem solving	Patient outcome improvement measures; Care quality indicators; Problem resolution effectiveness
Purposeful Orientation	Creative approaches to problem-solving valued and supported by leadership	Innovation behavior: Creative approaches to problem-solving v...	Innovation behavior scales; Performance measures; Behavioral assessments
Purposeful Orientation	Opportunity exploration including discovery, search, creation	Innovation behavior: Opportunity exploration including discov...	Innovation behavior scales; Performance measures; Behavioral assessments
Purposeful Orientation	Idea search based on existing knowledge sources in environment	Innovation behavior: Idea search based on existing knowledge ...	Innovation behavior scales; Performance measures; Behavioral assessments
Purposeful Orientation	Problem-solving approach addressing workplace challenges	Innovation behavior: Problem-solving approach addressing work...	Innovation behavior scales; Performance measures; Behavioral assessments
Purposeful Orientation	Addressing problems by assessing patient and nurse issues in care process	Patient care innovation	Care quality measures; Patient satisfaction; Clinical effectiveness indicators
Purposeful Orientation	Analyzing possible factors that cause emerging problems	Innovation behavior: Analyzing possible factors that cause em...	Innovation behavior scales; Performance measures; Behavioral assessments
Purposeful Orientation	Investigating phase identifying problems requiring technological solutions	Innovation behavior: Investigating phase identifying problems...	Innovation behavior scales; Performance measures; Behavioral assessments
Purposeful Orientation	Problem-solving skills and clinical decision-making abilities	Innovation behavior: Problem-solving skills and clinical deci...	Innovation behavior scales; Performance measures; Behavioral assessments
Purposeful Orientation	Innovation drivers addressing complex healthcare challenges	Patient care innovation	Care quality measures; Patient satisfaction; Clinical effectiveness indicators
Purposeful Orientation	Clinical environments pregnant with problems needing solutions	Innovation behavior: Clinical environments pregnant with prob...	Innovation behavior scales; Performance measures; Behavioral assessments
Purposeful Orientation	Well-defined problems versus ill-defined, complex, wicked problems	Complex Purposeful Orientation	Wicked problem recognition scale; Complex problem-solving measures; Systems thinking assessments
Purposeful Orientation	Innovation responding to obvious difficulties in healthcare delivery	Patient care innovation	Care quality measures; Patient satisfaction; Clinical effectiveness indicators
Purposeful Orientation	Innovation driven by obvious difficulty or challenging conditions	Innovation behavior: Innovation driven by obvious difficulty ...	Innovation behavior scales; Performance measures; Behavioral assessments
Purposeful Orientation	Purposeful Orientation addressing healthcare complexities	Patient care innovation	Care quality measures; Patient satisfaction; Clinical effectiveness indicators
Purposeful Orientation	Policy as mechanism to address contextual challenges	Innovation behavior: Policy as mechanism to address contextua...	Innovation behavior scales; Performance measures; Behavioral assessments
Purposeful Orientation	Policy implementation addressing nursing and healthcare problems	Patient care innovation	Care quality measures; Patient satisfaction; Clinical effectiveness indicators
Purposeful Orientation	Jobs to Be Done Theory focusing on solution-focused progress	Innovation behavior: Jobs to Be Done Theory focusing on solut...	Innovation behavior scales; Performance measures; Behavioral assessments
Purposeful Orientation	Solution-oriented approach to answer problems in nursing practice	Innovation behavior: Solution-oriented approach to answer pro...	Innovation behavior scales; Performance measures; Behavioral assessments
Purposeful Orientation	Innovation ability to solve problems and improve quality of care	Patient care innovation	Care quality measures; Patient satisfaction; Clinical effectiveness indicators
Purposeful Orientation	Nurses developing solutions to complex problems across healthcare	Patient care innovation	Care quality measures; Patient satisfaction; Clinical effectiveness indicators
Purposeful Orientation	Creative solutions to problems through continuous improvement processes	Innovation behavior: Creative solutions to problems through c...	Innovation behavior scales; Performance measures; Behavioral assessments
Purposeful Orientation	Innovation starting with good ideas that address identified needs	Innovation behavior: Innovation starting with good ideas that...	Innovation behavior scales; Performance measures; Behavioral assessments
Implementation	Application involves making innovations regular aspect of working processes	Innovation institutionalization	Implementation sustainability measures; Routine use assessments; Process adoption rates
Implementation	Implementation phase introducing new procedure and its use by department	Innovation deployment effectiveness	Implementation fidelity measures; Adoption success rates; Process integration assessments
Implementation	Idea championing with plans for implementation in unstructured environment	Innovation advocacy behavior	Champion behavior scale; Influence tactics inventory; Advocacy effectiveness measures
Implementation	Seven-phase technological creativity process ending in application and monitoring	Innovation behavior: Seven-phase technological creativity pro...	Innovation behavior scales; Performance measures; Behavioral assessments
Implementation	Prototyping phase designing and testing before standardization	Innovation development capability	Prototype quality measures; Testing effectiveness evaluations; Development milestone achievements
Implementation	Testing phase conducting clinical trials and revising based on results	Innovation behavior: Testing phase conducting clinical trials...	Innovation behavior scales; Performance measures; Behavioral assessments
Implementation	Applying phase disseminating technology to colleagues for practice implementation	Innovation behavior: Applying phase disseminating technology ...	Innovation behavior scales; Performance measures; Behavioral assessments
Implementation	Monitoring phase evaluating effectiveness and efficiency of technology	Operational efficiency innovation	Efficiency ratios; Productivity measures; Process improvement metrics
Implementation	Individual nurse or teams responsible for implementing innovation	Innovation behavior: Individual nurse or teams responsible fo...	Innovation behavior scales; Performance measures; Behavioral assessments
Implementation	Translation stage providing nurses with external and internal support	Innovation behavior: Translation stage providing nurses with ...	Innovation behavior scales; Performance measures; Behavioral assessments
Implementation	Process innovation building on organizational knowledge to improve services	Innovation behavior: Process innovation building on organizat...	Innovation behavior scales; Performance measures; Behavioral assessments
Implementation	Policy implementation as rolling out policies for optimizing health outcomes	Innovation behavior: Policy implementation as rolling out pol...	Innovation behavior scales; Performance measures; Behavioral assessments
Implementation	Policy dissemination communicating evidence to policymakers	Innovation behavior: Policy dissemination communicating evide...	Innovation behavior scales; Performance measures; Behavioral assessments
Implementation	Policy implementation research examining how policies are translated into practice	Innovation behavior: Policy implementation research examining...	Innovation behavior scales; Performance measures; Behavioral assessments
Implementation	Implementation strategies to adopt evidence-informed interventions	Innovation behavior: Implementation strategies to adopt evide...	Innovation behavior scales; Performance measures; Behavioral assessments
Implementation	Hybrid implementation designs evaluating policies and their impact	Innovation behavior: Hybrid implementation designs evaluating...	Innovation behavior scales; Performance measures; Behavioral assessments
Implementation	Assessment conducted throughout implementation phase for feedback	Innovation behavior: Assessment conducted throughout implemen...	Innovation behavior scales; Performance measures; Behavioral assessments
Implementation	Translation adoption of strategies leading to successful implementation	Innovation behavior: Translation adoption of strategies leadi...	Innovation behavior scales; Performance measures; Behavioral assessments
Implementation	Sustainability requiring balance between stability and evolution	Innovation behavior: Sustainability requiring balance between...	Innovation behavior scales; Performance measures; Behavioral assessments
Implementation	Prototyping to ensure innovative ideas are efficacious and effective	Innovation behavior: Prototyping to ensure innovative ideas a...	Innovation behavior scales; Performance measures; Behavioral assessments
Implementation	Dissemination and diffusion into practice through systematic processes	Innovation behavior: Dissemination and diffusion into practic...	Innovation behavior scales; Performance measures; Behavioral assessments
Implementation	Apply innovation to work through teamwork and reasonable support channels	Innovation behavior: Apply innovation to work through teamwor...	Innovation behavior scales; Performance measures; Behavioral assessments
Implementation	Workshop-based implementation with theoretical and practical components	Innovation behavior: Workshop-based implementation with theor...	Innovation behavior scales; Performance measures; Behavioral assessments
Implementation	Implementation of innovative roles through systematic competency development	Innovation behavior: Implementation of innovative roles throu...	Innovation behavior scales; Performance measures; Behavioral assessments
Implementation	Implementation essential for good ideas to become actual innovations	Innovation behavior: Implementation essential for good ideas ...	Innovation behavior scales; Performance measures; Behavioral assessments
Implementation	Product development journey from idea through commercialization	Innovation behavior: Product development journey from idea th...	Innovation behavior scales; Performance measures; Behavioral assessments
Implementation	Innovation Engine approach to systematic implementation across nursing	Innovation behavior: Innovation Engine approach to systematic...	Innovation behavior scales; Performance measures; Behavioral assessments
Value Creation	Personal and professional dynamism contributing to student innovation	Innovation energy and drive	Vitality scale; Professional engagement measures; Innovation motivation assessments
Value Creation	Professional inventiveness creating meaningful solutions for nursing	Innovation meaningfulness creation	Work meaning scales; Innovation impact significance; Professional fulfillment measures
Value Creation	Integration of innovation drivers aligning multiple factors for student success	Innovation behavior: Integration of innovation drivers aligni...	Innovation behavior scales; Performance measures; Behavioral assessments
Value Creation	Job productivity and lower levels of job burnout	Work performance optimization	Productivity indices; Maslach Burnout Inventory; Job performance evaluations
Value Creation	Job satisfaction and solving organizational problems	Organizational problem resolution satisfaction	Job satisfaction surveys; Problem resolution effectiveness; Organizational commitment scales
Value Creation	Organizational efficiency and effectiveness	Organizational performance enhancement	Efficiency ratios; Effectiveness indicators; Performance dashboards
Value Creation	Innovation leading to organizational competitive advantage	Strategic innovation advantage	Market position measures; Competitive advantage assessments; Strategic performance indicators
Value Creation	Caring for patients through solving health problems and maintaining safety	Patient care quality enhancement	Patient outcome measures; Care quality indicators; Patient satisfaction scores; Safety metrics
Value Creation	Caring for nurses by facilitating work and improving dignity	Nurse work experience improvement	Work environment scales; Nurse satisfaction surveys; Workflow efficiency measures
Value Creation	Caring for management through improving service quality and efficiency	Operational efficiency innovation	Efficiency ratios; Productivity measures; Process improvement metrics
Value Creation	Improved patient outcomes through nurse-led innovation	Patient outcome optimization	Clinical outcome measures; Patient safety indicators; Quality metrics; Mortality/morbidity rates
Value Creation	Opening access to care for underserved populations	Patient care innovation	Care quality measures; Patient satisfaction; Clinical effectiveness indicators
Value Creation	Creating systems to support health of elders living independently	Innovation behavior: Creating systems to support health of el...	Innovation behavior scales; Performance measures; Behavioral assessments
Value Creation	Transforming care at the bedside	Patient care innovation	Care quality measures; Patient satisfaction; Clinical effectiveness indicators
Value Creation	Patient wellness including reduced morbidity and mortality risk	Patient care innovation	Care quality measures; Patient satisfaction; Clinical effectiveness indicators
Value Creation	Half billion dollars more revenue over five-year period for innovative organizations	Financial performance impact	Revenue growth measures; ROI calculations; Financial performance indicators
Value Creation	Enhanced productivity, greater work engagement, increased wellbeing	Work productivity and engagement enhancement	Utrecht Work Engagement Scale; Productivity measures; Performance metrics
Value Creation	Positive impact on healthcare quality and patient safety	Patient safety enhancement	Patient safety indicators; Adverse event rates; Safety culture assessments
Value Creation	Meaningful everyday life for nursing home residents	Innovation behavior: Meaningful everyday life for nursing hom...	Innovation behavior scales; Performance measures; Behavioral assessments
Value Creation	Social innovation creating community connections and meaningful activities	Innovation behavior: Social innovation creating community con...	Innovation behavior scales; Performance measures; Behavioral assessments
Value Creation	Opening nursing homes to community creating fluid transitions	Innovation behavior: Opening nursing homes to community creat...	Innovation behavior scales; Performance measures; Behavioral assessments
Value Creation	Process change and new services or products delivering value	Innovation behavior: Process change and new services or produ...	Innovation behavior scales; Performance measures; Behavioral assessments
Value Creation	Revolutionary transformation through affordable, accessible solutions	Innovation behavior: Revolutionary transformation through aff...	Innovation behavior scales; Performance measures; Behavioral assessments
Value Creation	Innovation ability improvement by 10.7% through workshop intervention	Innovation behavior: Innovation ability improvement by 10.7% ...	Innovation behavior scales; Performance measures; Behavioral assessments
Value Creation	Innovation product scores increasing by 23.1% post-workshop	Innovation behavior: Innovation product scores increasing by ...	Innovation behavior scales; Performance measures; Behavioral assessments
Value Creation	Improved quality of care for patients through enhanced innovation abilities	Patient care innovation	Care quality measures; Patient satisfaction; Clinical effectiveness indicators
Value Creation	Innovation supporting vision, mission, and values of healthcare organization	Patient care innovation	Care quality measures; Patient satisfaction; Clinical effectiveness indicators
Value Creation	Continuous improvement embedded into normal work processes	Innovation behavior: Continuous improvement embedded into nor...	Innovation behavior scales; Performance measures; Behavioral assessments
Value Creation	Enhanced healthcare delivery through innovative nursing role implementation	Patient care innovation	Care quality measures; Patient satisfaction; Clinical effectiveness indicators

Value Creation	Innovation driving future creation of innovative healthcare ecosystems	Patient care innovation	Care quality measures; Patient satisfaction; Clinical effectiveness indicators
Value Creation	Interprofessional collaborations to transform healthcare systems	Patient care innovation	Care quality measures; Patient satisfaction; Clinical effectiveness indicators
Value Creation	Solving wicked problems transforming healthcare system	Complex Purposeful Orientation	Wicked problem recognition scale; Complex problem-solving measures; Systems thinking assessments
Value Creation	Enhanced productivity and greater work engagement through innovation	Work productivity and engagement enhancement	Utrecht Work Engagement Scale; Productivity measures; Performance metrics
Value Creation	Progress in nursing through solution-focused innovation approaches	Innovation behavior: Progress in nursing through solution-foc...	Innovation behavior scales; Performance measures; Behavioral assessments
Value Creation	Innovation contributing to organizational and societal improvement	Innovation behavior: Innovation contributing to organizationa...	Innovation behavior scales; Performance measures; Behavioral assessments
