# Supplementary material for: Nursing innovation: a comprehensive definition and framework for implementation
Source: BMC Nurs. 2026 Aug 6;25:1056. doi: 10.1186/s12912-026-05124-7 (PMC13729330; doi:10.1186/s12912-026-05124-7)
Supplement: Supplementary file 2 [file 12912_2026_5124_MOESM1_ESM.docx]

**Supplementary Table 1. Search Strings by Database and Overview of Internet Search**

**Pubmed – 1887 results**

(

("nursing innovation"[tiab]

OR nurs*[tiab] AND innovat*[tiab]

OR "health innovation"[tiab]

OR "healthcare innovation"[tiab]

OR ("innovation definition"[tiab])

)

AND

(definition[tiab] OR concept[tiab]

OR "defined as"[tiab] OR "scope note"[tiab]

OR typolog*[tiab] OR framework[tiab])

)

NOT

(trial[pt] OR randomized[tiab] OR RCT[tiab] OR feasibility[tiab])

**CINAHL – 2,172 results**

(MH "Innovation" OR TI innovation OR AB innovation OR TI innovat* OR AB innovat*)

AND

(TI nurs* OR AB nurs* OR MH "Nursing" OR MH "Nursing Practice"

OR "nursing innovation" OR "health innovation")

AND

(TI definition OR AB definition OR TI concept OR AB concept

OR TI typolog* OR AB typolog* OR TI framework OR AB framework)

**Web of Science – 5,347 results**

TS = (

(innovation OR innovat* OR "innovation definition" OR "innovation typology")

AND

(nurs* OR "health innovation" OR "clinical innovation"

OR "organizational innovation" OR "service innovation")

AND

(definition OR concept OR framework OR typolog* OR "concept analysis")

)

**Business Source Complete – 886 results**

TI (innovation OR innovat* OR "innovation definition" OR "innovation typology"

OR "service innovation" OR "organizizational innovation"

OR "disruptive innovation" OR "incremental innovation")

AND

AB (definition OR concept OR framework OR typolog* OR theory)

AND

(TI nurs* OR TI health* OR AB nurs* OR AB health*)

**Dictionaries Consulted - 5**

Merriam-Webster

Oxford English Dictionary

Cambridge Dictionary

American Heritage Dictionary

Etymology Online

Search terms used individually:
• “innovation”
• “health innovation”
• “nursing innovation”
• “novelty”
• “creativity”
• “invention”

**Government & Professional Org Sources (Google Search)**

Searches combined general innovation terms with organization names and nursing-specific phrases; Restricted to documents available in English (written or translated).

Search date: October 2025.

(e.g., ‘innovation definition site:who.int’, ‘health innovation framework government’, ‘nursing innovation definition professional organization’, ‘OECD innovation definition’, ‘ANA nursing innovation’)

**Hand Searches (digital)**

Sources:
• Google Scholar
• Reference lists of included articles
• Nursing innovation center websites
