# Supplementary material for: Nursing innovation: a comprehensive definition and framework for implementation
Source: BMC Nurs. 2026 Aug 6;25:1056. doi: 10.1186/s12912-026-05124-7 (PMC13729330; doi:10.1186/s12912-026-05124-7)
Supplement: Supplementary file 3 [file 12912_2026_5124_MOESM2_ESM.docx]

**Supplementary Table 2. Summary of Quality Assessment Scores for Included Articles per Smith & Morelius (2021) Criteria**

| **Source (Author, Year)** | **Epistemology**  **(4)** | **Pragmatic**  **(4)** | **Linguistic**  **(4)** | **Logical**  **(2)** | **Total Score (14)** |
| --- | --- | --- | --- | --- | --- |
| Albert (2018) | 4 | 3 | 4 | 2 | **13** |
| Argyropoulous (2021) | 3 | 4 | 4 | 1 | **12** |
| Asurakkody (2018) | 4 | 3 | 4 | 2 | **13** |
| Bahari (2021) | 4 | 4 | 4 | 2 | **14** |
| Baregheh (2009) | 4 | 4 | 4 | 2 | **14** |
| Barr (2021) | 4 | 4 | 4 | 2 | **14** |
| Beaudet (2023) | 4 | 4 | 4 | 2 | **14** |
| Budden (2019) | 4 | 3 | 4 | 2 | **13** |
| Crossan (2010) | 4 | 4 | 4 | 1 | **13** |
| Drach-Zahavy (2001) | 4 | 4 | 4 | 1 | **13** |
| Flessa (2021) | 4 | 4 | 4 | 1 | **13** |
| Gao (2022) | 3 | 4 | 4 | 1 | **12** |
| Garcia (2002) | 4 | 3 | 4 | 1 | **12** |
| Gilmartin (1999) | 4 | 3 | 4 | 1 | **12** |
| Glor (1997) | 4 | 3 | 4 | 1 | **12** |
| Goyal (2025) | 4 | 3 | 4 | 2 | **13** |
| Greenhalgh (2004) | 4 | 4 | 4 | 2 | **14** |
| Heydari (2023) | 3 | 4 | 4 | 1 | **12** |
| Hughes (2006) | 4 | 3 | 4 | 1 | **12** |
| Joseph (2019a) | 3 | 4 | 4 | 2 | **13** |
| Joseph (2019b) | 4 | 4 | 4 | 2 | **14** |
| Kaya (2015) | 3 | 3 | 4 | 1 | **11** |
| Klein (1996) | 4 | 4 | 4 | 2 | **14** |
| Leary (2024) | 4 | 4 | 4 | 2 | **14** |
| Omachonu (2010) | 4 | 4 | 4 | 2 | **14** |
| Roberts-Davis (1998) | 3 | 4 | 4 | 1 | **12** |
| Rogers (2003) | 4 | 4 | 4 | 2 | **14** |
| Rylee (2023) | 4 | 3 | 4 | 2 | **13** |
| Schumpeter (1934) | 4 | 4 | 4 | 2 | **14** |
| Slettebo (2021) | 3 | 4 | 4 | 2 | **13** |
| Snow (2019) | 4 | 4 | 4 | 2 | **14** |
| Stephens (2022) | 3 | 4 | 4 | 1 | **12** |
| Stout (2023) | 4 | 4 | 4 | 2 | **14** |
| Taylor (2017) | 4 | 4 | 4 | 2 | **14** |
| Thakur (2012) | 4 | 4 | 4 | 2 | **14** |
| Weberg (2009) | 4 | 3 | 4 | 4 | **13** |
| Younas (2025) | 4 | 3 | 4 | 1 | **12** |
