# Supplementary material for: Nursing innovation: a comprehensive definition and framework for implementation
Source: BMC Nurs. 2026 Aug 6;25:1056. doi: 10.1186/s12912-026-05124-7 (PMC13729330; doi:10.1186/s12912-026-05124-7)
Supplement: Supplementary file 4 [file 12912_2026_5124_MOESM3_ESM.docx]

**Supplementary Table 3. Dictionary Definitions and Etymology of Innovation vs Related Concepts**

| **Term** | **Definition (Merriam-Webster)** | **Etymology / Word History** |
| --- | --- | --- |
| **Creativity** | “The ability to bring into existence, to produce through imaginative skill”. ^1^ | Root from Latin creare “to make, bring forth”; early modern usage tied to artistic creation, idea generation^6^ |
| **Ideation** | “The capacity for or the act of forming or entertaining ideas.” ^2^ | Derived from idea + -ation (Latin suffix indicating action or process); first attested ~1805 in English usage.^7^ |
| **Innovation** | “A new idea, method, or device: novelty”; “the introduction of something new.” ^3^ | From mid-15thc Latin innovatio “restoration, renewal,” from innovare “to change; to renew,” from in(into) + novus (new).^8^ |
| **Invention** | “Something invented; a device, contrivance, or process originated after study and experiment … a product of the imagination.” ^4^ | Invention: from Latin invenire “to come upon, find,” where the emphasis is on discovery or origination of something not previously existing.^9^ |
| **Novelty** | “Something new or unusual; “the quality or state of being novel: newness.” ^5^ | From Anglo-French novelté, from novel “new”; first known usage ~14-15th century.^10^ |

**Supplementary References:**

^1^Merriam-Webster. (n.d.). *Creativity*. In *Merriam-Webster.com dictionary*. Retrieved September 17, 2025, from <https://www.merriam-webster.com/dictionary/creativity>

^2^Merriam-Webster. (n.d.). *Ideation*. In *Merriam-Webster.com dictionary*. Retrieved September 17, 2025, from [https://www.merriam-webster.com/dictionary/ideation](https://www.merriam-webster.com/dictionary/ideation?utm_source=chatgpt.com)

^3^Merriam-Webster. (n.d.). *Innovation*. In *Merriam-Webster.com dictionary*. Retrieved September 17, 2025, from [https://www.merriam-webster.com/dictionary/innovation](https://www.merriam-webster.com/dictionary/innovation?utm_source=chatgpt.com) [Merriam-Webster](https://www.merriam-webster.com/dictionary/innovation?utm_source=chatgpt.com)

^4^Merriam-Webster. (n.d.). *Invention*. In *Merriam-Webster.com dictionary*. Retrieved September 17, 2025, from [https://www.merriam-webster.com/dictionary/invention](https://www.merriam-webster.com/dictionary/invention?utm_source=chatgpt.com)

^5^Merriam-Webster. (n.d.). *Novelty*. In *Merriam-Webster.com dictionary*. Retrieved September 17, 2025, from <https://www.merriam-webster.com/dictionary/novelty>

^6^Harper, D. (n.d.). Creativity. In **Online Etymology Dictionary**. Retrieved September 17, 2025, from <https://www.etymonline.com/word/create>

^7^Harper, D. (n.d.). Ideation. In **Online Etymology Dictionary**. Retrieved September 17, 2025, from <https://www.etymonline.com/search?q=ideation>

^8^Harper, D. (n.d.). Innovation. In **Online Etymology Dictionary**. Retrieved September 17, 2025, from [https://www.etymonline.com/word/innovation](https://www.etymonline.com/word/innovation?utm_source=chatgpt.com)

^9^Harper, D. (n.d.). Invention. In **Online Etymology Dictionary**. Retrieved September 17, 2025, from <https://www.etymonline.com/search?q=invention>

^10^Harper, D. (n.d.). Novelty. In **Online Etymology Dictionary**. Retrieved September 17, 2025, from <https://www.etymonline.com/word/novelty>
