# Supplementary material for: Nursing innovation: a comprehensive definition and framework for implementation
Source: BMC Nurs. 2026 Aug 6;25:1056. doi: 10.1186/s12912-026-05124-7 (PMC13729330; doi:10.1186/s12912-026-05124-7)
Supplement: Supplementary file 5 [file 12912_2026_5124_MOESM4_ESM.docx]

**Supplementary Table 4. Model, Borderline, Related, and Contrary Cases for the Concept Analysis of Nursing Innovation**

**Model Case (Exemplifies all attributes)**

Policy Innovation. A nurse serving as a city council member identifies gaps in emergency response for unhoused populations during extreme heat events. Drawing on her clinical expertise and community partnerships, she authors and passes a municipal ordinance that mandates cooling stations staffed by nurses, supported by redirected public health funding. The initiative is nurse-led (nursing agency), introduces a novel solution in the form of citywide nurse-managed cooling infrastructure, is purposefully oriented toward equity and population health, and is implemented through legislative action and budget allocation. Early evaluation shows value creation with reduced heat-related emergency visits and improved access for vulnerable groups.

Role Innovation. During the COVID-19 pandemic there was a shortage of nurses available to hire for inpatient departments. To address these staffing shortages, a clinical operations nursing director with responsibility for the float pool developed a formal, structured, nurse residency program in collaboration with human resources, nursing professional governance structures, nursing leadership, and the nursing professional development team. The pilot year enrolled two nurses into the residency program, which demonstrated 100% retention rate of these new graduate nurses at 2 years tenure, and also reduced the vacancy rate in the float pool. This demonstrated nursing agency (nurse-led), contextual novelty (normally nurses within that health system were not allowed to work in the float pool until they have one or more years of experience), purposeful orientation (addressing a nursing need), implementation (nurses were enrolled into the residency program), and value creation (reducing open, unfilled positions).

**Borderline Case (Ambiguous application of attributes)**

Process Innovation. A senior nurse executive was a member of the advisory board of a health-tech startup that markets a wearable fitness tracker called “CarePath Fit.” Her team named her Clinical Integration Lead for a 6-month pilot in three community clinics. She co-designed a nurse-managed care pathway that paired the tracker’s step data with weekly coaching scripts, materials in both English and Spanish, and a risk flag for inactivity. Once designed, the nurse retired from her role and was no longer involved in the work, while a project manager (non-nursing) took over the project for implementation. During the same window, the product team pushed out a software update to the fitness tracker that included gamification. At 6 weeks post-implementation, clinics noted small gains in average weekly steps and a minor drop in missed follow-ups, with most engagement attributed to the app’s gamification update. As a result, the clinic could not attribute effects from the nurse-authored elements. No workflow changes were adopted in the EHR, and the pathway was not routinized post-pilot. The fitness tracker product was implemented and generated value, but the nurse’s leadership was absent during the implementation period, and the simultaneous app update diluted the link between the nurses’ program design and the results, making this a borderline case rather than a model of nursing innovation.

Technology/Process Innovation. Nurses led education about alarm fatigue and piloted an artificial intelligence (AI)-based alarm management platform in an inpatient unit. They tracked alarms per patient day and patient experience scores about a restful environment for 12 weeks. At the end of the study, the alarm management software tool produced no clinically meaningful change in alarm volume or patient experience scores. After the pilot period, the nurses introduced a new pre-ventricular contraction (PVC) alarm threshold for low-risk patients (from 6 to 20 per minute) which did produce a statistically significant and sustained change in alarm volume. This borderline case meets nursing agency, contextual novelty, purposeful orientation, and implementation, but value creation was marginal. The PVC change demonstrated that improvement was possible, but it was a separate process and cannot be credited to the AI-based innovation. As a result, this could be labeled as “nursing innovation in development” or “emerging nursing innovation” because it has not yet met the full value-creation threshold.

**Related Case (Shares similarities but lacks one defining attribute)**

Product Innovation. A nurse manager pilots the use of an existing electronic whiteboard system to improve shift-to-shift handoffs in an intensive care unit. The system is already in use in other departments, but the nurse adapts it slightly to include nursing-specific data fields. The project demonstrates **nursing agency**, purposeful orientation, implementation, and value creation through improved communication, but the **novelty** is limited since the technology is not new and the adaptation is incremental.

Education Innovation. The Education Department purchases an off-the-shelf virtual reality (VR) sepsis recognition module. A staff nurse facilitates sessions and handles scheduling. The nurse cannot alter learning objectives, scenarios, scoring, or LMS integration. The module runs during two quarterly skills fairs. Attendance is high, and learner satisfaction improves. Pre- to post-quiz scores rise modestly. Unit metrics tied to practice do not change, including time to sepsis bundle activation and lactate draw times. This example is a related case because it includes nurses in a facilitator role (no elements of agency in design or implementation), while also having minimal impact on the unit of adoption.

**Contrary Case (Clearly not nursing innovation)**

Marketing/Communications Innovation. A hospital’s marketing department designs a new billboard campaign to advertise its emergency services. Nurses are photographed in the ads but have no role in developing the concept, shaping the messaging, or approving the materials. The campaign is novel in appearance and widely disseminated, but it reflects a **vendor-led initiative** with **no nursing agency**, no purposeful orientation toward advancing nursing values, and no evidence of value creation beyond generic publicity. Because nurses are only used symbolically and not as decision-makers, this example is a marketing activity, but it is **not nursing innovation.**

Process/Organizational Technology Innovation. A hospital rolls out a new electronic documentation template created entirely by the information technology (IT) department to standardize nursing notes. Nurses are required to use it but had no role in conceiving, designing, or piloting the tool. Although the change is implemented and may create value through efficiency and compliance, it lacks nursing agency and is not novel from the perspective of nursing practice. This is an example of organizational change imposed on nursing, but it does not qualify as nursing innovation.
